# PhysiBoSS: a multi-scale agent-based modelling framework integrating physical dimension and cell signalling

**DOI:** 10.1093/bioinformatics/bty766

**Published:** 2018-08-30

**Authors:** Gaelle Letort, Arnau Montagud, Gautier Stoll, Randy Heiland, Emmanuel Barillot, Paul Macklin, Andrei Zinovyev, Laurence Calzone

**Affiliations:** 1 Institut Curie, PSL Research University, Paris, France; 2 INSERM, U900, Paris, France; 3 CBIO-Centre for Computational Biology, MINES ParisTech, PSL Research University, Paris, France; 4 Université Paris Descartes/Paris V, Sorbonne Paris Cité, Paris, France; 5 Gustave Roussy Cancer Campus, Villejuif, France; 6 INSERM, U1138, Paris, France; 7 Equipe 11 Labellisée par la Ligue Nationale Contre le Cancer, Centre de Recherche des Cordeliers, Paris, France; 8 Intelligent Systems Engineering, Indiana University, Bloomington, IN, USA

## Abstract

**Motivation:**

Due to the complexity and heterogeneity of multicellular biological systems, mathematical models that take into account cell signalling, cell population behaviour and the extracellular environment are particularly helpful. We present PhysiBoSS, an open source software which combines intracellular signalling using Boolean modelling (MaBoSS) and multicellular behaviour using agent-based modelling (PhysiCell).

**Results:**

PhysiBoSS provides a flexible and computationally efficient framework to explore the effect of environmental and genetic alterations of individual cells at the population level, bridging the critical gap from single-cell genotype to single-cell phenotype and emergent multicellular behaviour. PhysiBoSS thus becomes very useful when studying heterogeneous population response to treatment, mutation effects, different modes of invasion or isomorphic morphogenesis events. To concretely illustrate a potential use of PhysiBoSS, we studied heterogeneous cell fate decisions in response to TNF treatment. We explored the effect of different treatments and the behaviour of several resistant mutants. We highlighted the importance of spatial information on the population dynamics by considering the effect of competition for resources like oxygen.

**Availability and implementation:**

PhysiBoSS is freely available on GitHub (https://github.com/sysbio-curie/PhysiBoSS), with a Docker image (https://hub.docker.com/r/gletort/physiboss/). It is distributed as open source under the BSD 3-clause license.

**Supplementary information:**

Supplementary data are available at *Bioinformatics* online.

## 1 Introduction

Mathematical modelling of individual cells has already been widely used to address questions tackling the complexity of biological systems (Mogilner *et al.*, 2006; Ventura *et al.*, 2006). Due to the high inter-dependency of the different biological scales driving the development of cells (and tumours in the case of cancer), models that explore the interplay between cells and their environment are also needed to better describe tumourigenesis (Anderson and Quaranta, 2008; Deisboeck *et al.*, 2011; Loessner *et al.*, 2013; Sanga *et al.*, 2007). As a result, multi-scale modelling is being recognized as an important contribution to build a comprehensive mechanistic view of cancer (Deisboeck *et al.*, 2011; Loessner *et al.*, 2013), to enable predictions at the level of the cell, the population of cells (e.g. the tumour) or the organ.

The dynamical study of cell populations is crucial to improving prognosis or treatment efficiency (Sanga *et al.*, 2007). Knowing the rules governing the behaviour of each separate component in a population is not enough to predict the emergent behaviour of such a complex system, and similarly, understanding the disregulated processes in an individual cell is not enough to predict the behaviour of the cell population. The most famous cellular automaton model, Conway’s Game of Life, demonstrates how simple single-cell rules that are perfectly known can generate non-intuitive and complex behaviours at the multi-cellular systems level.

Some studies have demonstrated that genotypically identical cells can adopt different phenotypes according to their environment (Gligorijevic *et al.*, 2014) or tumourigenic factors (Leal-Egaña *et al.*, 2017). Notably, the interplay between spatial position and signalling is critical in development, for example in morphogenesis (Nelson *et al.*, 2006), in cell competition (Levayer *et al.*, 2016) and in cell fate decisions through Notch signalling patterning (Liao and Oates, 2017). The interaction between all these different factors is also crucial for exploring the diverse modes of cell motility (Friedl and Wolf, 2010) and is thus a core question in understanding diseases such as cancer. Computer modelling is therefore increasingly necessary to tackle such complex problems (Sharpe, 2017).

Several mathematical formalisms can be used to model both the individual cell and population levels (e.g. discrete, continuous, hybrid, etc.) (Le Novère, 2015; Loessner *et al.*, 2013; Tanaka, 2015). The choice of appropriate formalism depends on the modelling scope (e.g. see the summary of different models used to study shape homeostasis in Gerlee *et al.*, 2017). At the level of the cell population, some previous modelling efforts have been done with cellular automaton models (e.g. Altinok *et al.*, 2011), with cellular Potts models (e.g. Kumar *et al.*, 2016) or centre-based models (e.g. Drasdo and Höhme, 2005; Ramis-Conde *et al.*, 2009). These approaches have typically focused on the emergent multicellular dynamics after assigning simple, microenvironment-driven single-cell phenotypes, rather than including both the intracellular events and how individual cellular alterations might affect the population.

There have been some attempts to couple intracellular and population dynamics. However, to overcome computational problems, such models usually needed to be kept simple, or were limited in size. Note that simple models of the signalling pathways with small numbers of variables inside each cell can already be very informative, as for example the model in Bekkal Brikci *et al.* (2008) that used partial differential equations to explore the transition from one cell cycle phase to another at the population level, or the model with ordinary differential equations (ODEs) to explore population dynamics (Rué and Garcia-Ojalvo, 2013). Nevertheless, to take the microenvironment into account, some crucial components need to be added to these frameworks, and the models can quickly become very complex.

Quite interestingly, Gao *et al.* (2016) also demonstrated the necessity of taking into account intracellular dynamics in the population dynamic to study CD8+ T-cell response to external stimulati. Their multi-scale on-lattice approach (Prokopiou *et al.*, 2014), combining a model of ODEs within a Cellular Potts model, provides a complete and powerful model that was computationally expensive, depending on the size of the intracellular model. Other multi-scale models have been developed to answer specific questions in the last few years (e.g. Anderson *et al.*, 2006; Belmonte *et al.*, 2016; Gerlee and Anderson, 2009; Jeon *et al.*, 2010; Ramis-Conde *et al.*, 2009), but they are usually specifically tailored to a given problem, and cannot be adapted in a straightforward manner to new questions. Additionally, very few of the software packages are available as open source, thus hindering repeatability, reproducibility and community-driven refinement. Other tools such as EpiLog have been developed to include multilevel logical models in a 2D lattice representing the epithelium (Varela *et al.*, 2018). Currently, EpiLog supports intracellular models of great size with a lattice composed of up to a million cells, but do not yet fully model the growth and movements of the cell population. Other 2D developments have been published that bridge intracellular pathways and cell population interactions, such as VirtualLeaf (Merks *et al.*, 2011) that uses an off-lattice vector-based framework of irregular shapes to depict plant leaves’ cells, and Athale and Deisboeck (2006)’s work that uses an off-lattice agent-based method to simulate the effects of epidermal growth factor receptor (EGFR) density and activation on tumour growth dynamics.

Agent-based models are particularly suitable methods for a multi-scale approach, allowing modellers to integrate multiple scales as well as spatial considerations, and providing a mostly intuitive representation of biological systems (An *et al.*, 2009). Importantly, these models are also very flexible, allowing the simulation of a wide range of situations with minor adaptations.

In order to simulate not only populations of isolated cells but also organized groups of cells (tissues, organoids, etc.), cell-centred, off-lattice models are an appropriate choice of agent-based models (Osborne *et al.*, 2017; Tanaka, 2015). Among the available tools implementing cell-centred agent-based models, CellSys (Drasdo and Höhme, 2005), Chaste (Mirams *et al.*, 2013) and PhysiCell (Ghaffarizadeh *et al.*, 2018; Macklin *et al.*, 2012) are particularly interesting. The implementation of physical laws in CellSys reproduces multi-cellular phenomena quite accurately (Drasdo *et al.*, 2007; Hoehme and Drasdo, 2010). However, the multi-scale model developed by this group was restricted to differential equation models in a particular scenario (Ramis-Conde and Drasdo, 2012; Ramis-Conde *et al.*, 2008; Schluter *et al.*, 2014) and thus difficult to adapt to other biological questions. Moreover, CellSys to date has not been released as open source. Among other available tools, Chaste provides an environment to implement different kinds of modelling approaches (agent-based, cellular automaton, vertex model, etc.) within the same framework (Mirams *et al.*, 2013; Pitt-Francis *et al.*, 2009) as well as ODEs for intra-cellular modelling. Importantly, its implementation allows the direct comparison of outputs from the different modelling techniques and outlines their advantages and limits (Osborne *et al.*, 2017). However, due to all these possibilities, it is a more complex environment than other agent-based tools. PhysiCell is an agent-based software with the particular advantages of being open source and having minimal dependencies. Nevertheless, this software still lacked intra-cellular modelling.

Recently, promising agent-based open source software, such as MecaGen (Delile *et al.*, 2017) and EmbryoMaker (Marin-Riera *et al.*, 2016), showed the power of combining mechanical behaviour and gene regulation to understand embryogenesis and could be used to study other developmental problems. However, the gene regulatory networks are modelled with ODEs, which might restrict it to relatively small signalling representations and raises the difficulty of parameter estimation. Moreover, due to its morphogenesis scope, MecaGen does not consider cell division dynamics (cell volume growth, death, etc.), or issues such as clonal diversity. All of these software tools have been summarized and can be compared in Supplementary Table S1.

We choose to start from an agent-based-dedicated open source software, PhysiCell, and, since we wish to link intra- and inter-cellular descriptions, we combine it with a tool for modelling Boolean networks, MaBoSS. Our ultimate goal is to develop an open source flexible modelling framework that combines the description of the signalling pathways inside individual cells and their interaction with the environment.

To explore and integrate cell population dynamics, cellular signalling mechanisms, and the interplay between cells and their surrounding (i.e. other cells or the microenvironment), we propose to combine an agent-based approach with a Boolean representation of biochemical events taking place in each cell. For that purpose, we have developed a new software framework, PhysiBoSS, that combines and extends two well-established tools: a signalling pathway modelling tool, MaBoSS (Stoll *et al.*, 2012, 2017), which performs stochastic simulations of the signalling pathway inside each cell, and an agent-based modelling tool, PhysiCell (Ghaffarizadeh *et al.*, 2018), that represents each individual cell as a physical dynamical entity.

We detail our implementation of PhysiBoSS and demonstrate its use with a model of cell-fate decisions in response to Tumour Necrosis Factor (TNF) to illustrate the importance of considering cell–cell communication in homogeneous as well as heterogeneous cell populations. With this cancer example, we will showcase the use of PhysiBoSS to numerically study the effect of treatment regimes on a heterogeneous cell population and its effects on clonality and tumour growth.

## 2 Materials and methods

To address the issue of including individual cell description into an agent-based model, we adapted, merged and expanded two existing open source software tools. The first one, PhysiCell (Ghaffarizadeh *et al.*, 2018), focuses on the evolution of a multicellular system (particularly tumours) by simulating the dynamics of a population of cells under specific constraints, in 2 D or in 3 D. The second one, MaBoSS (Stoll *et al.*, 2012, 2017), defines a continuous-time Markov process on the state transition graph of a Boolean model. Note that a state transition graph is a graph which encompasses all possible transitions between model states, and a model state is a vector which captures the activity of all the nodes of the model. MaBoSS estimates the probability of visiting reachable states of the model. The two software tools were merged so that the conditions for the tumour’s growth depend on the status of individual cells and the behaviour of individual cells is influenced by their environment.

### 2.1 PhysiCell

PhysiCell is an open source agent-based software with minimal dependencies. To improve computational efficiency, it simulates off-lattice position and volume but not morphology; where needed, cell morphology is approximated as a soft sphere. The code is parallelized using OpenMP when possible, allowing the simulation of thousands of cells for several days in a reasonable time (a few hours), and simulations of 10^5^ to 10^6^ cells can be run over several days. An efficient implementation of the diffusion of environmental entities (oxygen, glucose, growth factors, etc.) and their interaction with the cells (uptake, secretion, etc.) is also provided by PhysiCell’s BioFVM module (Ghaffarizadeh *et al.*, 2016). Beyond secretion and uptake of diffusing substrates, PhysiCell has implemented key phenotypic behaviours: cell volumetric growth, adhesion, repulsion, directed and random motility, cell cycle progression and death processes. PhysiCell allows users to attach tailored C++ functions and data structures to each individual cell, which can then modify the cell agents’ phenotypes dynamically throughout a simulation. We use this functionality to add MaBoSS’ signalling model to each individual cell agent, and then to link each agent’s signalling state to its phenotypic behaviour.

### 2.2 MaBoSS

MaBoSS (Stoll *et al.*, 2012, 2017) is an open source C++ simulator of Boolean models of signalling pathways. In this logical modelling framework, variables (genes, proteins or specific protein functions) can take two values, 0 or 1, mimicking their activity. Each variable is updated according to the status of its regulating variables, connected by logical connectors AND, OR and NOT. Variable state transitions are stochastically calculated from parametrisable rates. MaBoSS can simulate a Boolean model describing the signalling pathways inside an individual cell and providing probabilities of reaching the stable states. Inputs can be upstream events such as receptor activation, and outputs correspond to phenotypic behaviours such as cell death, proliferation, migration, etc. The optimized implementation of this formalism allows for computation of a high number of variables in the network.

### 2.3 PhysiBoSS

**Fig. 1.**
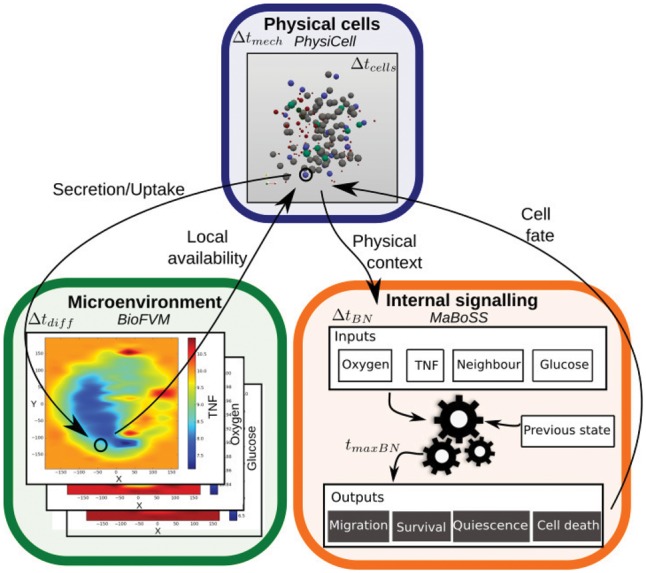
Schematic representation of PhysiBoSS. Three main parts are interconnected: the microenvironment representation in BioFVM (green, bottom left), allowing simulation of diffusing entities; the physical representation of cells as dynamic spheres in PhysiCell (blue, top); and the signalling modelling of each cell in MaBoSS (orange, bottom right) (Color version of this figure is available at *Bioinformatics* online.)

PhysiBoSS integrates these two software frameworks to obtain a detailed description of each cell’s behaviour and how an alteration in a cell can affect the whole population. There are three main parts in the PhysiBoSS structure:

- BioFVM module handles the simulation of one or more diffusing environmental entities (Ghaffarizadeh *et al.*, 2016). It simulates diffusion, degradation and release of diffusible entities in the extracellular space, including extracellular matrix (ECM) (Fig. 1, green). Space is discretised in a voxel mesh containing information of the local density of the modelled diffusing entities (oxygen, glucose, growth factors, etc.);
- PhysiCell core handles the representation of the cells’ mechanics (Ghaffarizadeh *et al.*, 2018) and key phenotypic behaviours. A cell is represented as a soft sphere with two radii: cellular and nuclear. It can move and interact with neighbouring objects, divide, and change its properties according to specific conditions (Fig. 1, blue);
- MaBoSS core computes the solutions of a logical model representing the dynamics of a network of intracellular events (Stoll *et al.*, 2012). This module gathers its input conditions from the PhysiCell core evaluation (e.g. presence of neighbours or of growth factors, etc.) and retrieves outputs that correspond to cell fates in PhysiCell core (e.g. forming adhesions, migrating, or dying). The logical model and parameter descriptions are defined in two files following MaBoSS standard, so any MaBoSS model can be directly used in PhysiBoSS, provided that its inputs and outputs are integrated in the agent-based part (Fig. 1, orange).

Importantly, as this system involves a broad range of events at different biological scales, PhysiBoSS uses different time steps for different parts of the model (see Supplementary File S1).

#### 2.3.1 Numerical implementation

The main core of the software is adapted from PhysiCell, and the MaBoSS module is compiled as a linked external library. PhysiBoSS is written in C++ with minimal external dependencies. PhysiBoSS uses one executable file reading an associated specific parameter file. This structure is convenient to generate numerous simulations with different parameters/configurations. Three executables are provided:

- **PhysiBoSS**, the main executable, requires four files: a model specific parameter file, the two MaBoSS model files, and a file with initial conditions. If the simulation is going to model ECM, then a matrix-specific initial condition file is needed.
- **PhysiBoSS**_**CreateInitTxtFile** generates an initial condition file that specifies the cells’ initial positions and their volumes for a variety of classic geometries (e.g. sphere, cylinder or rectangle). For more complex geometries (e.g. *Hello World* example in the PhysiBoSS GitHub documentation), the initial configuration can be created from a binary image of the desired shape by placing cells on the positive areas.
- **PhysiBoSS**_**Plot** generates an .svg output snapshot of the simulation at a given time point (more details on the wiki). Note that we plan to develop further visualization tools and a graphical interface in future releases of PhysiBoSS.

The details for preparing, executing and visualizing a simulation can be found in detail in Supplementary File S1 and scripts are provided on the GitHub repository to automate them, along with step-by-step examples with all the necessary files. The computational time required for one individual run is strongly sensitive to its parameters, such as time/space steps, number of cells, diffusing entities, etc. (Supplementary Table S2).

#### 2.3.2 PhysiBoSS features

PhysiBoSS works with spherical **cells** that represent living cells that can grow/shrink, divide, move, interact with their environment or other cells and die. These cells progress through the **cell cycle** and change their physical properties, have a front-rear **polarity** and can be part of **cell strains**, where each cell shares a set of common physical and genetic parameters (Supplementary File S1).

**Simulation of different cell strains**—Users can simulate heterogeneous populations of genetically and/or physically different cells. For this, the parameter file must take into account all physical parameters of each strain type, as well as the transition rates of mutated genes of genetically different strains. PhysiBoSS implements mutation by modifying each variable’s on–off transition rates, rather than changing the Boolean network structure. For example, over-expression of a gene will be implemented as a node with very high activation rate and a null deactivation rate. These transition rates need to be controlled through a variable in MaBoSS configuration files, and their values need to be specified for each cell strain in the parameter file. (See GitHub repository for more details and examples.)

**Extracellular matrix representation**—As PhysiBoSS aims to integrate environmental, multicellular and intracellular descriptions of biology, the representation of the ECM was addressed in this framework. In previous theoretical works, ECM has been represented by a fibrous matrix in a mechanochemical model (Ahmadzadeh *et al.*, 2017), as ‘cells’ of a Cellular Potts Model (Kumar *et al.*, 2016), as linear elastic medium (Bischofs and Schwarz, 2003), as a network of Hookean springs (Zhu and Mogilner, 2016), as (non-)deformable objects composed of networks of springs (Tozluoğlu *et al.*, 2013), or as passive spheres (Drasdo and Höhme, 2005). The choice for these different representations is strongly dependent on the biological question: a discrete ECM representation can be enough, while in other cases, it is necessary to model the deformation, softening, hardening or degradation of the ECM. This choice is also often a compromise between computational cost and the desired level of precision.

**Fig. 2.**
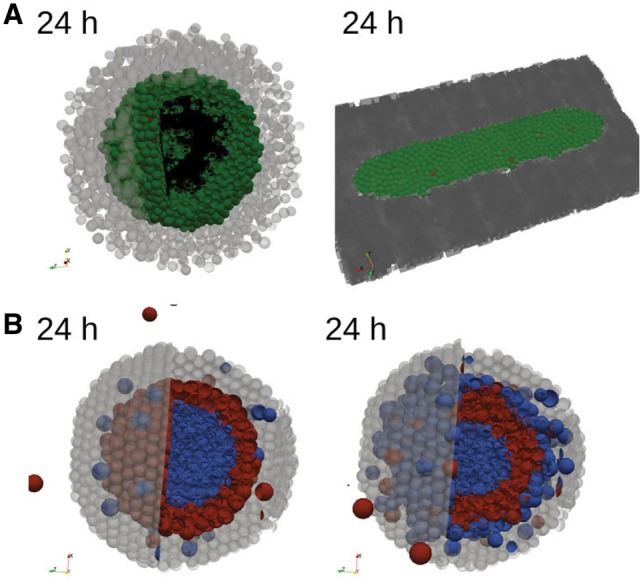
Examples of PhysiBoSS features. (A) Final state (24 h) of a simulation of active cells (green, *Survival*; black, *Necrosis*; red, *Apoptosis*) spheroid inside a core of passive ECM agents (light grey) (left panel). Final state (24 h) for active cells inside a fixed ECM dark grey field (right panel). (B) Final images (24 h) of mechanical cell sorting surrounded by ECM (passive spheres, light grey): the blue cell line (inside) forms strong junctions, while the red cell line (extern) is weakly adhesive. Cells do not adhere to ECM (left panel), or only the blue cell line can attach to the matrix (right panel). (A–B) Initial states of the simulations are shown in Supplementary Figure S1 (Color version of this figure is available at *Bioinformatics* online.)

PhysiBoSS proposes two ways of implementing ECM modelling: the first representation is to use ECM as passive spheres (Fig. 2A) that can be pushed by other spheres or active cells depending on a friction coefficient. Cells can also degrade (or reinforce) these passive spheres upon contact with user-defined rates, by decreasing (or increasing) the radius of the passive spheres. The advantage of this implementation is that it integrates well within PhysiCell code structure and is not highly expensive computationally. This approach can be used, for example, to model the ability of cells to create tracks in the ECM or to simulate steric hindrance due to Dextran presence in a medium (Delarue *et al.*, 2014). However, its precision is poor and not very well suited for simulations of filamentous environment. Moreover, if the simulated space is large (or spheres are small), the high number of necessary passive spheres can drastically increase the computational cost.

The second representation uses the BioFVM module by considering ECM as a non-diffusing density. Cells can interact with the surrounding matrix by adherence, repulsion, degradation and deposition of ECM (Supplementary File S1), but they cannot push it. This allows for a finer spatial ECM definition with small mesh sizes. This representation is very convenient to describe a non-deformable matrix and could be used for example to study cell population growth on restricted areas, as micropatterns (Fig. 2B). However, its non-elastic formulation can be a major drawback for other studies.

**Cell–cell and cell–matrix adhesions**—The core modelling of cell–cell and cell–matrix interactions from Macklin *et al.* (2012) are maintained in PhysiBoSS, with slight modifications to allow dynamic evolution of homotypic, heterotypic (Duguay *et al.*, 2003) and matrix adhesions. Notably, a coefficient of cadherin/integrin densities involved in the adhesion was included to respond to the (de-)activation of the Boolean network’s adhesion pathway, so that this coefficient varies accordingly to reflect the different protein recruitment. Differences in strength between these diverse adhesions can be sufficient to drive specific cell sorting (Steinberg, 1963). To validate our implementation, we verified that our framework reproduced the sorting behaviour explored in Cerchiari *et al.* (2015). The results of this can be seen in Figure 2B, where the test was limited to a purely mechanical-driven sorting. However, PhysiBoSS could be used to further explore cell sorting by taking into account cell proliferation and differences in motility, which have been seen to impact the sorting mode or efficiency (Strandkvist *et al.*, 2014).

## 3 Results

We showcase three examples of scientific problems that PhysiBoSS can address, using a cell fate model upon TNF injection as a case study. This Boolean model describes the pathways leading to the main cellular fates in response to TNF receptor activation: *Survival* (read-out of proliferative cells), *Apoptosis* and non-apoptotic cell death (*NonACD*) (Calzone *et al.*, 2010). Earlier simulations of this model using MaBoSS predicted that isolated cells in a fixed system lead to heterogeneous fate commitment (Calzone *et al.*, 2010), and this heterogeneity could be interpreted as the limited efficiency of TNF treatment on tumours. The model used here has been slightly modified from Calzone *et al.* (2010). We included nodes that account for the mRNAs of some of the components (Supplementary Fig. S2A), which introduce some delays between protein-to-protein interactions and signal transduction on one side and translation and transcription time-frames on the other side, as we set lower probability rates for transcription and translation events (Mahaffy and Pao, 1984) (Supplementary Fig. S4F). Indeed, previous mathematical models showed it is necessary to account for the delay in transcription (Lewis, 2003; Mahaffy, 1988; Momiji and Monk, 2009) to explain observed biological behaviours (Hirata *et al.*, 2002; Shimojo *et al.*, 2008).

Simulating the model using PhysiBoSS let us address important questions related to collective behaviours (such as homogeneous or heterogeneous populations), spatial (diffusion and consumption of TNF, paracrine secretion of TNF from neighbouring cells, etc.) and dynamical behaviours (continuous or discontinuous presence of TNF, autocrine secretion of TNF through NFκB’s feedback loop, etc.). We were also able to test the effect of clones in a tumour and their response to TNF treatment. Different TNF dose regimes in homogeneous cell populations were first simulated, using as initial conditions proliferating and healthy functioning cells (Supplementary File S2). At frequent intervals, each cell’s internal signalling model was updated according to its current environment (TNF internalization or not) and its current signalling state (resulting from MaBoSS previous iterations). This determined the cell (de-)activation of TNF-α secretion, through NFκB feedback, and ultimately the cell fate decision (switch the cell state either to *Survival*, *Apoptosis* (irreversible), or *NonACD* (irreversible), Supplementary Fig. S2B).

### 3.1 Model validation

To validate our model, we referred to two studies focusing on different TNF treatment regimes using 3T3 mouse fibroblast cells in microfluidic chambers (Kellogg *et al.*, 2015; Tay *et al.*, 2010). These works show that cells’ response to TNF injection is highly heterogeneous. The proportion of cells that responded to TNF injection within the first 8 h on average (reported in their experiments as transient relocation of NFκB to the nucleus) depended on the dose concentration and the duration of the injection, referred by Tay *et al.* as ‘stimulus area’ (Tay *et al.*, 2010). The fraction of responding cells varied from 0, for a dose area smaller than 10^2^ ng s/mL, to a ‘total’ response when dose area was around 10^4^ ng s/mL, with a Hill-like dependency on the stimulus area: Hill coefficient around 1.5 and a 20–50 min response time (Kellogg *et al.*, 2015).

**Fig. 3.**
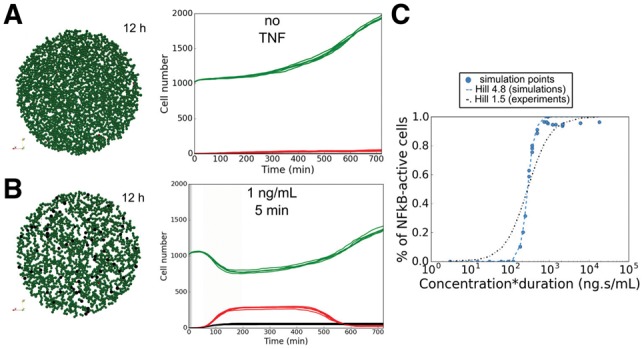
Population response to TNF injection. (A) Simulation without TNF. Snapshot of a simulation after 12 h (left). Time evolution of the number of cells in each cell fate (right) for five simulations. (B) Same as A for a low-dose injection of TNF (1 ng/mL during 5 min). (C) Fraction of ‘activated’ cells (transient NFκB activation) compared to the initial number of viable cells according to TNF stimulus area (concentration time duration). The blue dotted line represents the Hill-function fit to the simulation data (coefficient 4.8), and the black dashed line represents a Hill-function of coefficient 1.5 as in experiments from (Kellogg *et al.*, 2015). (A–C) Green, *Proliferative* cells; red, cells committed to *Apoptosis*; black, cells committed to *NonACD*. Initial disk radius: 400 µm (Color version of this figure is available at *Bioinformatics* online.)

We first calibrated our model to simulate growth dynamics of 3T3 cells. In the absence of TNF, the population grows without constraints (Fig. 3A, Supplementary Fig. S3A), with a doubling time of approximatively 16 hours (Kim *et al.*, 2004). We then explored the response of the population when TNF was injected in the medium (details on TNF dynamics in Supplementary File S2). Upon limited TNF injection, only a partial response of the population was observed, in accordance with the experimental observations described above (Fig. 3B, Supplementary Fig. S3B). We then varied both the TNF concentrations and the injection durations and obtained a similar Hill-like dependency of the fraction of active cells to injection area (Fig. 3C). Parameters of TNF dynamics were chosen to have similar range of response to similar injections’ doses of experimental data, ranging from no response under 10^2^ ng.s/mL to a ‘total’ response above 10^3^ ng s/mL (Hill coefficient: 4.8, 40–60 min response time).

We measured the activated fraction of cells present at the end of the simulation (i.e. when NFκB got activated at least transiently), as presented in the experiments. In our simulations, 20% of the cells internalized TNF and committed to *Apoptosis* without activating NFκB pathway. In fact, Tay *et al.* showed that after 8 hours of high TNF concentration (10 ng/mL), cells started to express less anti-apoptotic genes and more pro-apoptotic genes (Tay *et al.*, 2010), in qualitative agreement with the simulation predictions.

As shown in Figure 3C, the simulated response was stiffer than the experimental one, although the model reproduced qualitatively the observed behaviour within the same range of values. This suggests that our model can be used to predict qualitatively the cell population response to TNF in other conditions, and that the simulations can retrieve a range of TNF concentration values and percentage of cells that responded, but one should be cautious before interpreting the results quantitatively.

### 3.2 Multicellular spheroid response to TNF treatment

*In vitro* multi-cell spheroid models are now widely used to study tumourigenesis (Loessner *et al.*, 2013; Sutherland, 1988), due to their similarity with *in vivo* conditions, allowing for a good compromise between system complexity and clinical relevance (Hirschhaeuser *et al.*, 2010). Our model was used to investigate how a multi-cell spheroid would respond to TNF injection, showing that it could be used to test the effect of injection frequency, clonality or complex heterogeneous scenarios.

**Fig. 4.**
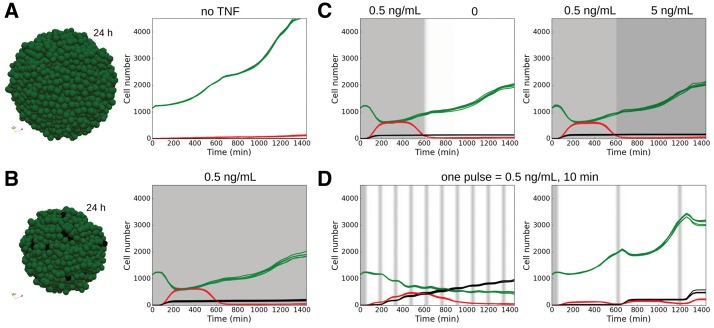
Spheroid response to TNF injection. (A) Simulation in the spheroid model without TNF. Snapshot of a simulation (left) after 24 h. Time evolution of the number of cells in each cell fate (right) for five simulations. (B) Same as A for a continuous low-dose injection of TNF (0.5 ng/mL, continuously). (C) Simulation when TNF injection (0.5 ng/mL) is stopped (left) or drastically increased (5 ng/mL, right) after 600 min. Time evolution of the number of cells in each cell fate for five simulations under each condition. (D) Effect of pulse injection frequencies in the model simulations. Time evolution of the number of cells in each cell fate for five simulations when pulsed injections (0.5 ng/mL during 10 min) are repeated every 150 (left) and 600 (right) min. (A–D) Green, *Proliferative* cells; red, *Apoptosis*; black, *NonACD*. Grey shading represents TNF injection. Initial spheroid radius is 100 µm (Color version of this figure is available at *Bioinformatics* online.)

In the absence of TNF, the spheroid grew as cells doubled their volumes and divided (Fig. 4A, Supplementary Fig. S4). Continuous injection of a low dose of TNF drastically reduced the expansion of the population (a 4.5-fold increase in cell numbers after 24 h in the non-treated simulation, compared to a 1.8-fold increase in the treated one), because ∼50% of the initial population committed to *Apoptosis* or *NonACD* in response to TNF (Fig. 4B, Supplementary Fig. S4). However, cells that activated the survival NFκB pathway became resistant to TNF (*Survival* stable state) and transmitted this resistance to the daughter cells, who inherit their mother cell’s signalling network state. This sub-population continued to grow independently of the TNF presence: discontinuing the TNF injection or increasing it 10-fold after 600 min did not affect the overall behaviour (Fig. 4C). In the first 600 min, these cells received a constant external input (TNF activation) and reached a stable state, as could be predicted from a MaBoSS simulation of an individual cell (Calzone *et al.*, 2010). In the first scenario, increasing the TNF dose did not affect the signalling network of these already activated cells or their stable state. In the second scenario proliferative cells were still present as, due to the absence of TNF, cells switched from a NFκB pathway-activated proliferative stable state to an un-activated proliferative stable state.

From these results, we hypothesized that injections of short duration of TNF (instead of having a continuous regime) could sensitize the cells to TNF. To test that idea, we simulated pulses of TNF injections at given frequencies and found out that transient exposure did strongly affect the population’s response (Fig. 4D, Supplementary Fig. S4C). This is important to consider as *in vivo* tissue cells are subjected to bursts of TNF expression from neighbouring immune response cells, while they might be under continuous injection in *in vitro* trials. Cells that were proliferative after the first injection were still responding to TNF in the following injections and the proportion of dying cells was much higher than with a continuous treatment, showing the importance of considering non-steady regimes. Note that this conclusion was also valid in 2D simulations (data not shown), which suggests that these behaviours were not strongly affected by the different TNF diffusions due to the geometries of the simulations, monolayer or spheroids, so long as sufficient TNF reached all the cells. The predicted differential response between transient and continuous exposures had been observed in a recent *in vitro* study (Lee *et al.*, 2016), where the authors observed more death for short 1 min pulses compared to long 60 min pulses in Hela cells. Their results suggested that the duration of the pulse has an effect on the differential activation of the pro-apoptotic or pro-survival pathways.

Notably, cells activated the apoptotic pathway in response to the first injection, whereas later injections committed cells mostly to *NonACD* (Fig. 4D), thus highlighting the importance of dynamics in the cells’ responses. This was consistent with the construction of our network, with faster *Apoptosis* commitment (Supplementary File S2). Moreover, changing the transcription rate in the model affected the type of cell fate decision, as increasing it favoured necrosis (*NonACD*) (Supplementary Fig. S4F). Indeed, faster transcription caused, among other effects, faster mXIAP activation that caused *Apoptosis* inhibition and faster mROS production of ROS, benefiting *NonACD* cell fate.

This suggests that PhysiBoSS can be used to screen frequencies and concentrations of treatment injections, narrowing *in vitro* investigations. Furthermore, it is also possible to test the system’s response in different cell types, with different TNF secretion rates in response to NFκB activation, which would affect the overall sensitivity to TNF concentrations (Supplementary Fig. S4D). We also used the tool to test the effect of the initial spheroid size on the TNF availability, the overall effect on global multicellular behaviour; initial tests using different ranges did not yield substantially different results (Supplementary Fig. S4E).

### 3.3 Response to TNF treatment of heterogeneous multi-cellular spheroids

**Fig. 5.**
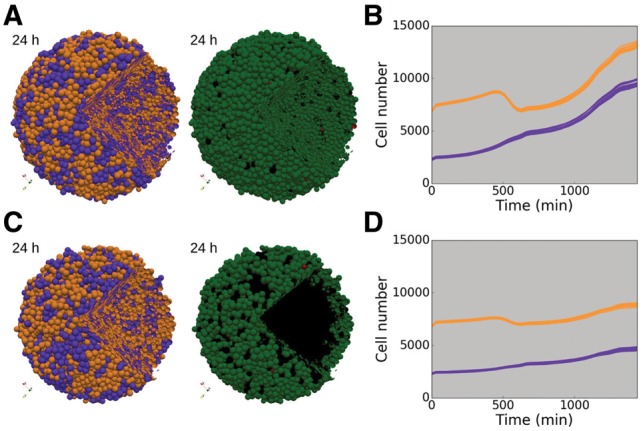
Genetically heterogeneous population under TNF treatment. Simulations of heterogeneous population composed of 75% of WT cells (orange) and 25% of IKK+ and cFLIP+ mutated cells (purple). (A) Snapshots of a genetically heterogeneous population simulation at initial and final time (24 h), with cells coloured by cell type (left and middle) or by cell fate (right). (B) Time evolution of the number of cells in each strain (WT and mutated) for 10 simulations. Grey shading indicates presence of TNF in continuous injection at 0.5 ng/mL. (C) Same as A with oxygen dynamics taken into account. (D) Same as B for simulations with oxygen diffusion. (A–D) Cell fate colours: green, *Proliferative* cells; red, *Apoptotic*; black, *NonACD*. Initial spheroid radius: 200 µm, + stands for over-expression (Color version of this figure is available at *Bioinformatics* online.)

One major challenge in pharmacological targeted treatments is the high level of spatial and temporal heterogeneity (Dagogo-Jack and Shaw, 2017), within the population due to the presence of different clones that respond differentially to the same conditions. To illustrate the effect of treatment on a genetically heterogeneous population, we simulated a spheroid initially composed of 75% of wild type strain (non-mutated, WT) and 25% of mutated cells with over-expressed(+) IKK and cFLIP (Fig. 5A). This double mutation was found to drastically promote cell survival using our pipeline of computational tools for logical models exploration (Montagud *et al.*, 2017) (Supplementary File S2). As expected, part of the WT population died under TNF treatment while the mutant population survived and proliferated (Fig. 5B). Importantly, the presence of the mutated population did not impact the response of the WT population: the final ratio of surviving WT cells compared to their initial number was similar to the one in a WT-only population (Supplementary Fig. S5C, no significant difference under Kolmogorov–Smirnov test). This phenomenon was also observed with two other mutations promoting either *Apoptosis* or *NonACD*, or with different initial proportion of WT cells in the total population (Supplementary Fig. S5).

In this simulation, cell communication was limited to TNF consumption/secretion and physical interaction. However, it is known that in a crowded environment such as a tumour, there is cell competition for resources like oxygen, nutrients and growth factors. To study the impact of resource competition among cell strains, we included oxygen diffusion and its cell consumption in the 3D spheroid set-up. A threshold under which cell commits to necrosis (*NonACD*) due to lack of oxygen was fixed (Supplementary File S2, Parameter table). As a consequence, in a homogeneous WT population without TNF, a necrotic core formed with a thin proliferative rim around it (Supplementary Fig. S6A), as described for large spheroids (Freyer and Sutherland, 1986; Sutherland, 1988). Under TNF treatment, the homogeneous population growth was strongly limited by the combination of TNF- and oxygen-mediated death (Supplementary Fig. S6B).

When mixed with the IKK+ and cFLIP+ mutated population, WT cells had to compete with proliferating mutant cells for oxygen in addition to surviving TNF signalling. Thus, the majority of the WT cells that did not commit to *Apoptosis* or *NonACD* by TNF signal, committed to *NonACD* by lack of access to oxygen (Fig. 5C and D). WT strain growth was considerably reduced compared to its growth in the homogeneous spheroid (Supplementary Fig. S6E). Similarly, when mixed with pro-*Apoptotic* or pro-*NonACD* mutants, WT cells had better access to oxygen and proliferated more than in a homogeneous spheroid (Supplementary Fig. S6C–E). This competition among strains was also observed for other ratios of WT/mutated populations (Supplementary Fig. S6F).

The resulting tumour is the consequence of the different adaptive abilities of these strains to the environmental conditions and to the TNF treatment. As illustrated here, this may result in the growth of resistant clones that gain access to nutrients. Importantly, it was recently shown that the tumour spatial structure strongly impacts the adaptive therapy efficiency (Bacevic *et al.*, 2017). Hence, using tools such as PhysiBoSS that include both spatial distribution and signalling networks is necessary for exploring and predicting the best clinical adaptive therapy strategies (Gatenby *et al.*, 2009).

## 4 Discussion

PhysiBoSS provides a way to bridge gene perturbations to cell population dynamics, taking into account microenvironmental perturbations. It is thus a unique tool to address issues as clonality in tumours, taking into account both intra-clonal (through stochasticity within MaBoSS and PhysiCell) and inter-clonal heterogeneity, the interaction of individual cells with the microenvironment (TNF, oxygen, etc.), and their temporal evolution (Dagogo-Jack and Shaw, 2017). In particular, PhysiBoSS allows showcasing a substantial difference of the cell population’s response to perturbations in TNF availability, which proves to be dependent on the signalling pathway dynamics (see also Lee *et al.*, 2016).

Multi-scale models have a great potential to study morphogenetic events by combining biochemical patterning with cell signalling and mechanics (Delile *et al.*, 2017; Marin-Riera *et al.*, 2016). Indeed, the overall population-level organization can be influenced both by cell differentiation in response to external signals (e.g. growth factor access) and cell organization by mechanical clues (e.g. differences in adhesion or motility). Computational tools such as PhysiBoSS can be used to predict the resulting organization of such interplay between genetic and phenotype factors under environmental perturbations, and thus reduce experimental exploration (Sharpe, 2017).

One limitation of PhysiBoSS is its spherical representation of cells morphology. In the next version of PhysiBoSS, we plan to propose other shapes such as an ellipsoidal shape as in Delile *et al.* (2017) and cylindrical shape as in Marin-Riera *et al.* (2016). We also plan to test PhysiBoSS using high-performance computing, similarly to the approach presented by Coulier and Hellander (2018), as well as high-throughput investigations on HPC resources as in Ozik *et al.* (2018). Additionally, we will extend its representation of the extracellular matrix, so that users can choose different modes of implementation according to the biological questions. Indeed, PhysiBoSS will be modified so as to offer different levels of representations from a very abstract representation (as currently possible as a field or passive spheres), to a more realistic representation (e.g. filamentous environment, which could be done by introducing a finite element mesh for the ECM, as suggested in Ghaffarizadeh *et al.*, 2018). Finally, another direction could be to combine MaBoSS with other agent-based modelling software, to allow for different frameworks (e.g. Cellular Potts, vertex model) according to the biological question of interest.

PhysiBoSS is available on GitHub (https://github.com/sysbio-curie/PhysiBoSS), under the BSD 3-clause license, with its own DOI (10.5281/zenodo.1194827). PhysiBoSS is compatible with most Unix systems and a Docker image (https://hub.docker.com/r/gletort/physiboss/) allows one to run PhysiBoSS on incompatible systems. We provide in the repository the source code of PhysiBoSS, several scripts to facilitate its use, reproducible examples (with all necessary files), and extended documentation on its wiki.

## Funding

This work received funding from the European Union Horizon 2020 research and innovation program (grant agreement No 668858, PrECISE project). It is part of the Chemotaxis project, funded by ‘Programme HTE’ from ITMO Cancer, in collaboration with ITMO BCDE and ITMO Technologies pour la santé from the ‘alliance nationale pour les sciences de la vie et de la santé (AVIESAN)’ with Institut National du Cancer and Inserm in the framework of Plan Cancer. AM was partly funded by INVADE grant from ITMO Cancer. PM and RH were partly supported by the Breast Cancer Research Foundation and the Jayne Koskinas Ted Giovanis Foundation for Health and Policy.

## Supplementary Material

Supplementary File S1

Supplementary File S2

Supplementary Information
