# Supplementary material for: PhysiBoSS: a multi-scale agent-based modelling framework integrating physical dimension and cell signalling
Source: Bioinformatics. 2018 Aug 30;35(7):1188–96. doi: 10.1093/bioinformatics/bty766 (PMC6449758; doi:10.1093/bioinformatics/bty766)
Supplement: Supplementary File S1 [file bty766_supplementary_file_s1.pdf]

Supplementary File 1: PhysiBoSS implementation details

Supplementary for PhysiBoSS: a multi-scale agent based modelling framework integrating physical dimension and cell signalling

Gaelle Letort, Arnau Montagud, Gautier Stoll, Randy Heiland, Emmanuel Barillot, Paul Macklin, Andrei Zinovyev, Laurence Calzone

Contents

1 PhysiBoSS general use **2**
1.1 Preparing simulations . . . 2
1.2 PhysiBoSS inputs . . . 2
1.3 Output . . . 3

2 PhysiBoSS implementation details **4**
2.1 Cell class implementation . . . 4
2.2 Cell-cell adhesions . . . 6
2.3 Cell-ECM adhesions . . . 6

3 Agent-based - MaBoSS interface **8**
3.1 Inputs of the Boolean model potentially considered in PhysiBoSS . . . 8
3.2 Outputs of the Boolean model potentially considered in PhysiBoSS . . . 10
3.3 Network state . . . 11
3.4 Time scales . . . 11

1 PhysiBoSS general use

1.1 Preparing simulations

Even though a single PhysiBoSS simulation can be easily prepared and run manually, it is very convenient to do this automatically for more extensive computations. We propose the use of script files to allow for multiple runs of one simulation set-up in the folder `scripts/presimu` of the GitHub repository (https://github.com/gletort/PhysiBoSS/). This customized version of the python script pre-config (Nedelec, 2017) performs automatic generation of simulation parameter files or initial files from a common template. For each parameter file created, a sub-folder of the main simulation is automatically created (run0, run1, run2...) containing the necessary input files and output sub-folders. This allows the user to generate different simulations while varying only one or few parameters, based on a unique common parameter template file. Then, each simulation is run independently and results can be directly compared to assess the effect of the varied parameter(s).

1.2 PhysiBoSS inputs

As mentioned in the main text, it was decided to build PhysiBoSS as a single executable, and to read input files to specify the current simulation set-up. Users can specify a parameter file, an initial cells configuration file, an initial extra-cellular matrix (ECM) configuration file and/or MaBoSS network configuration files. Default values are provided when one file or parameter is not specified.

The files needed for a proper PhysiBoSS run are:

- **Parameter file**: this is an XML file, as proposed in the MultiCellDS standardization initiative (see `multicellds.org`) that defines all the parameters required for a simulation. To parse the XML files, we integrated in our code the Tinyxml2 scripts, freely available at `http://www.grinninglizard.com/tinyxml2/index.html`. In the current version, we have 4 main parts: a set of parameters defining the simulation features (time steps, spatial limits, etc.), a set of parameters defining the cell properties (one for each cell strain to simulate), the definition of the network parameters and a set of parameters to define the initial configuration (either a geometry specification or initial file name). If a required parameter does not have a user-defined value in this file, a default value will be used. The complete list of parameters and their default values used are written in the output properties.xml file.

- **Initial file**: a semi-colon-delimited TXT file, containing in each line the position, volume and state information of one cell. The structure of this file is the same as the output files so one output file from a previous simulation can be used as an initial configuration for another simulation. Such an initial file can be automatically created by an executable (PhysiCell_CreateInitFile) developed within present work, that specifies the simulation's geometrical set-up in an XML file (e.g. a sphere of radius 100 μm composed of cells of average 10 μm radius, and surrounded by passive spheres). An additional script is provided to be able to create more complex initial geometries by using the positive area coordinates of binary images (script available on the Wiki).

- **Network files**: the Boolean network instance needs 2 files, one that specifies the network structure and one that specifies the initial conditions and simulation parameters. Those files are the same as the ones used in a standalone MaBoSS simulation. Thus any Boolean network from other studies can be used in a PhysiBoSS simulation, provided that the input and output nodes of the network are in the PhysiBoSS framework (link to section of PC-MB connectors).

- **ECM file**: similarly to the initial cells positions, it is also possible to specify the initial ECM spatial distribution using a semi-colon-delimited TXT file. This file must contain the voxel positions containing ECM with a local density values associated to those voxels.

Those input files must be placed in the main folder of the simulation, which contains also two sub-folders for the simulation output files (Fig SI1).

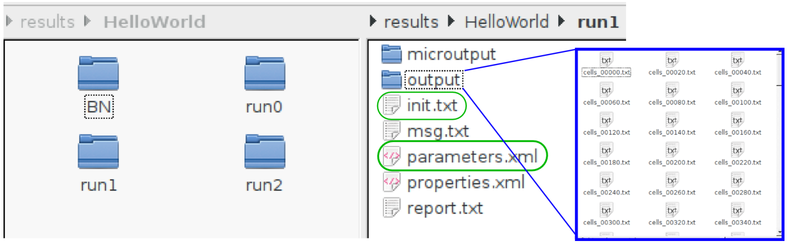

Fig 1. Organization of the files part of a typical simulation. Example of how simulation folders and files are organized to run PhysiBoSS. In this example, the parent directory of the simulation contains the folder `BN` with the network files in it, common to all sub-simulations. Then 3 sub-simulation folders are used to simulate similar configuration with, in this case, one parameter changing in between the different runs.

1.3 Output

A typical run of PhysiBoSS generate two kind of files: summary files and data files. The summary files consist in 1) a XML file containing the properties of the simulation (parameter values) that have been used in current simulation, and 2) a report TXT file that print an one-line summary of the number of cells that divided, died, average amount of densities present, and wall time passed in between two output time points.

The other file type, the data files, is a series of TXT files that contain the simulation results (position and state of cells) that are regularly written at a given user-defined time interval in the `output` sub-folder created in the main folder of the current simulation (Fig SI1).

To visualize the time evolution of these simulation results, we used the software Paraview which allows high flexibility and interactive viewing options. State files configuration files are available in the GitHub repository to set-up the visualization from the `TXT` files, which can also be easily customized if needed (Fig SI2). We also described in the documentation all the steps to set up the visualization from scratch without using the state files.

Additionally, PhysiBoSS can also generate `SVG` snapshot files at a given time points (or at a chosen frequency) during the simulation. This option was already available on PhysiCell and its use is depicted in the GitHub Wiki with detailed examples (https://github.com/gletort/PhysiBoSS/wiki).

Another information that is also written in `TXT` files is the density values of the environment elements that are stored in the `microutput` sub-folder. These files bear density values of each voxel at each time step, so they can easily be quite heavy. An option not to write all the voxels values but only a given percentage is proposed to have a lower-resolution approximation of the ECM values. As with the TXT files that defined cells' properties, the frequency of writing these files can also be adjusted to avoid writing many heavy files. These files can also be visualized with Paraview and one state file allowing to overlay ECM representation and cells is available.

`Python` scripts that read directly the output `TXT` files were used to analyse multiple simulations at once and are available in the GitHub repository (https://github.com/gletort/PhysiBoSS/tree/master/scripts/postsimu).

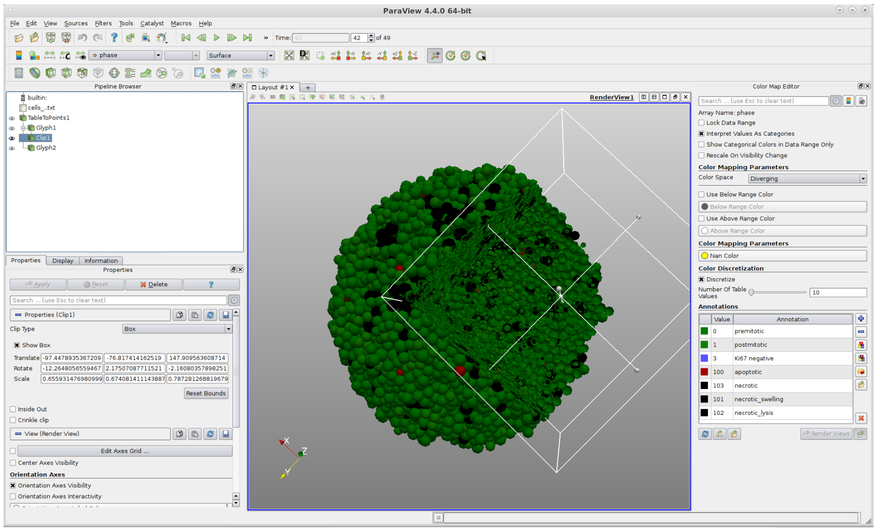

Fig 2. Visualization with Paraview using our state file. Example of how a simulation can be visualized, using the software Paraview, and our state file proposed to set it up (sphere_quarter.pvsm).

2 PhysiBoSS implementation details

2.1 Cell class implementation

We briefly present here the model of a cell in PhysiBoSS. Full implementation documentation is also available within the release, in the `doc` folder.

The system is described by a set of agents, which can be either passive or active cells (Fig 3). A passive cell cannot move but can be pushed by neighbouring cell. Its volume is fixed, except if a contacting active cell degrades it, reducing its radius. In our framework, these passive cells have been used to depict microenvironment particles. Note that this is one of the two ways that we have considered microenvironment, the other being the use of the `BioFVM` module to consider microenvironment as a not-diffusing density.

An active cell is how our framework considers living cells: they can grow/shrink, divide, move, interact with its environment or with other cells, and die. Active cell cycle progression is handled by a CellCycle class to determine the different phases of the cell and their transition. Boolean network control of the cell's proliferation is also integrated in derived class of CellCycle. A cell also has a set of properties, like its level of polarization, adherence, motility, etc. that can vary during the simulation as they are connected to the outputs of the signalling network. Finally, common properties that do not vary of all cells part of the same cell strain are shared in a CellLine object for computational efficiency.

Definition of an active cell in PhysiBoSS

In order to integrate them in our multi-scale framework, PhysiBoSS cell features have been modified and expanded from PhysiCell ones (Ghaffarizadeh *et al.*, 2018). The resulting features of an individual cell in PhysiBoSS are:

- **Cell**: Active cells represent the living cells that can grow/shrink, divide, move, interact with its environment or with other cells, and die. They are restricted to a spherical shape and contains a spherical nucleus surrounded by cytoplasm. Additionally, sets of common properties of cells are defined, we refer to them as "cell strains".

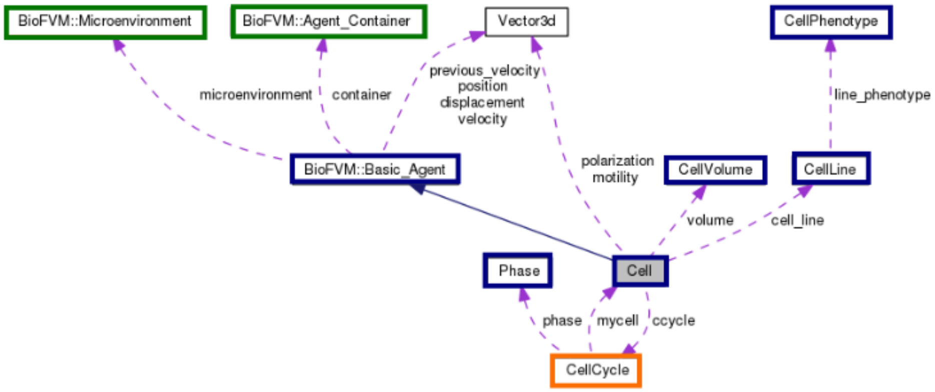

Fig 3. Implementation of a cell in PhysiBoSS. Adapted from the Doxygen documentation generation. Green rectangles represent part of the code dealing with the microenvironment (BioFVM), blue ones for the "physical" cells implementation (PhysiCell) and orange ones for the network computation (MaBoSS).

- **Cell cycle progression**: This features captures the cell's changes in physical properties along its cell cycle progression. During each phase of its cell cycle, cells can have different dynamics (phase duration, cell volume changes, etc) and mechanical properties (cell adhesiveness, motility, etc). In PhysiBoSS, rates of phase transitions are determined stochastically or deterministically, according to environmental conditions (e.g. oxygen access), as was defined in PhysiCell, and they can also be determined by the individual Boolean model evaluation. This implementation depends on the communication between the agent-based evaluation and the inputs of the Boolean network, and the communication between the outputs of the Boolean network and their effect on cell behaviour. Description of present network inputs/outputs and their feedback with the agent-based part are discussed later.

- **Cell strain**: Each cell strain shares a set of common physical parameters: adhesion parameters, default motility parameters, oxygen dependency parameters, cell cycle progression dynamics and mechanical properties, etc. Each cell strain also shares a set of genetic values, so different mutants will be part of different strains. Notably, most genetic variations will have different physical parameters (e.g. a cell with no *E-cadherin* will lack cell-cell adhesion), but there could be genetic variations that have no physical effect as well as physical parameters changes not rooted in genetic variations.

- **Cell polarity**: In the context of PhysiBoSS, only front-rear polarity is considered as it is usually associated with directed mesenchymal migration (Etienne-Manneville, 2008). The extent of cell polarization is determined by p_c coefficient between 0 (not polarized) and 1 (fully polarized). The resulting polarity axis is used to bias cell motility by the addition of an active motile force and to determine the division axis by using its perpendicular (Haeger *et al.*, 2014; Théry and Bornens, 2006), with a randomness degree if the cell is not strongly polarized (following PhysiCell model (Ghaffarizadeh *et al.*, 2018)). Cell polarity axis direction is updated according to the velocity-alignment model to take into account cells re-polarization in response to environmental clues. Cells align their polarity direction p with their current velocity v, with a certain persistence (τ) depending on the cell type (Albert and Schwarz, 2016; Basan *et al.*, 2013; Camley and Rappel, 2014; Szabó *et al.*, 2010):

$$\dot{p} = v - \frac{1}{\tau} p$$

Note that this equation is integrated with a semi-implicit scheme in the code. These cell polarity considerations can be particularly important in the context of collective cell migration (Hakim and Silberzan, 2017), or in morphogenesis as highlighted in a multi-scale 2D lattice-based model (Gerlee *et al.*, 2017).

2.2 Cell-cell adhesions

The implementation proposed in PhysiCell in Macklin *et al.* (2012) was modified to integrate more easily homotypic and heterotypic cell-cell adhesions, which can have different strengths especially if different cadherins are involved (Chu *et al.*, 2004; Labernadie *et al.*, 2017; Takeichi, 1991). Repulsion when cells overlap is calculated as:

$$\nabla\psi(\mathbf{r}) = ccr(c_1, c_2) \begin{cases} -(c\frac{|\mathbf{r}|}{R_N} + 1)\,\frac{\mathbf{r}}{|\mathbf{r}|}, & 0 \leq |\mathbf{r}| \leq R_N, \\ -(1 - \frac{|\mathbf{r}|}{R})^2\,\frac{\mathbf{r}}{|\mathbf{r}|}, & R_N \leq |\mathbf{r}| \leq R, \\ \mathbf{0} & else \end{cases} \tag{1}$$

with c_1, c_2 the two interacting cells, R_N the sum of their nuclear radii, R the sum of their radii, $\mathbf{r}$ the differences of their respective positions, $c = (1 - \frac{R_N}{R})^2 - 1$. Hence, repulsion is stronger if the nuclei overlap as they are much less deformable than the cell's cytoplasm. We assumed that the repulsion strength between the two cells is determined by the most repulsive one ($ccr(c_1, c_2) = max(ccr_1, ccr_2)$), where the ccr_i coefficients are the repulsive force for each cell strain (if they are more or less deformable).

Attraction, caused by cadherin-mediated adhesion, is calculated as :

$$\nabla\varphi(\mathbf{r}) = cca(c_1, c_2) \begin{cases} -(1 - \frac{|\mathbf{r}|}{R_{max}})^2\,\frac{\mathbf{r}}{|\mathbf{r}|}, & 0 \leq |\mathbf{r}| \leq R_{max}, \\ \mathbf{0} & else \end{cases} \tag{2}$$

with R_{max} the maximal distance where cells are considered to be able to interact, which is a little above the sum of their radii to take into account for their deformability and protrusions formation. We assumed that the adhesion strength is limited by the weakest of the two cells: $cca(c_1, c_2) = min(cca_1^{ho}, cca_2^{ho})$ if cells are of the same strain, $cca(c_1, c_2) = min(cca_1^{he}, cca_2^{he})$ for heterotypic interactions. The value of the coefficients cca_i^j can vary between two bounded values according to the cell current recruitment of cadherins:

$$cca_i^j = cca_i^j(min) + (cca_i^j(max) - cca_i^j(min)) * p_{adh} \tag{3}$$

where p_{adh} is the current percentage of the cell adhesiveness, whose value is controlled by the network's output.

2.3 Cell-ECM adhesions

The representation of the interactions between the cell and the extra-cellular matrix depends on the choice of the ECM modelling, that can be as passive spheres or as fixed densities. In both cases however, we calculated the repulsive/adhesive forces between a cell and a neighbouring ECM component, in a similar way to the interactions between two cells. ECM is considered as a sphere (of given radius in the passive spheres representation, and half-diagonal value of the voxel in the density case), which has a coefficient of interactive strength (local ECM density) c_{ecm}. The "nuclear" radius of the ECM "sphere" is the same as the total radius of the ECM and its maximal interactive distance is also limited to the radius, as it is not deformable. The cell-ECM interaction evaluation is the same as the cell-cell case, with the repulsive coefficient: $cer(c_1, ecm) = c_{ecm} * cer_1$, cer_1 being the cell strain coefficient of repulsion from the ECM, and

the adhesive coefficient: $cea(c_1, ecm) = c_{ecm} * cea_1$, with cea_1 the current strength of cell adhesion to the matrix. Similarly to cca_i^j, the adhesive coefficient of the cell to the matrix varies between two bounded values $cea_i(min)$ and $cea_i(max)$ according to a coefficient of how much integrins are recruited in the cell p_{ecm}:

$$cea_i = cea_i(min) + (cea_i(max) - cea_i(min)) * p_{ecm} \tag{4}$$

3 Agent-based - MaBoSS interface

PhysiBoSS handles the communication between a ”physical” agent-based representation of individual cells with their signalling pathways, modelled by MaBoSS framework. An important step to combine those two software is defining the interface between inputs and outputs of the Boolean network with the agents (Fig 4).

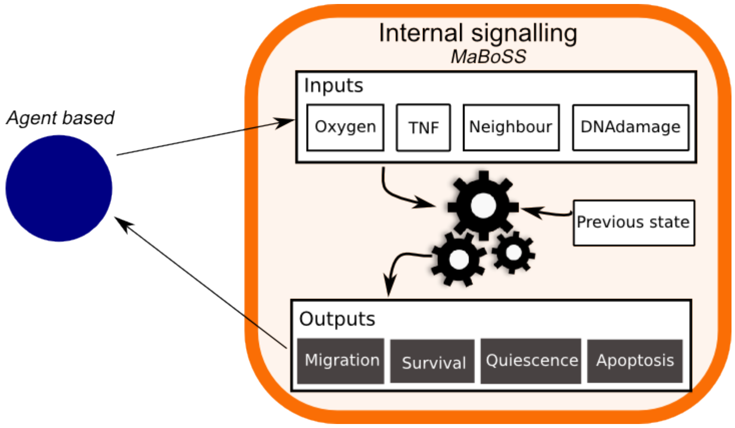

Fig 4. Schematic representation of the PhysiBoss' interface between PhysiCell and MaBoSS for each individual cell.

Here we present the list of possible Boolean network inputs handled by the agent-based part of the software. For each input node, we have also explained how the value of this node is calculated by the agent-based part. Then a list of Boolean network output nodes that can be retrieved and used as feedbacks in the agent-based model are also presented. For each possible output node's value, the agent-based part defines a behaviour in response, which we briefly present in this list.

3.1 Inputs of the Boolean model potentially considered in PhysiBoSS

- **Neighbours** : Represents if cell has active spheres as neighbours, passive cells (ECM) are not considered.
  Values: 1 if cell has enough contact with neighbour cells (compared to a given threshold $contact\_cell > contact\_cc\_threshold$, contact of cell = overlap of cells radii / cell radius), 0 otherwise.
  Parameter dependency: *contact_cell_cell_threshold* in cell_properties section of Parameters file. Note that potentially there will be one *contact_cell_cell_threshold* for each cell strain.

- **Nei2** : Represents if cell has mature adherens junctions that prevent the cell from moving.
  Values: 1 if cell has enough contact with neighbour cells (compared to higher threshold: two times the neighbour threshold $contact\_cell > 2 * contact\_cc\_threshold$, contact of cell = overlap of cells radii / cell radius), 0 otherwise.
  Parameter dependency: *contact_cell_cell_threshold* in cell_properties (common to cell strain) parameters.

- **EGF, IGF TNF, GF, TGFBeta**: Represents growth factors (GF) presence, different types of GFs are possible. The corresponding GF must be present in the microenvironment entities (or node value will be 0). GFs densities in the microenvironment are calculated by the BioFVM module and can vary according to diffusions, cell uptakes/secretions and external source addition.

Values: 1 if local density of the given GF is high enough (above a given threshold, defined by e.g. *GF_threshold*), 0 otherwise. If users want to have an active GF receptor without simulating the GF's microenvironment distribution, they can define *GF_threshold* as -2. Parameter dependency: *GF_threshold* in cell_properties section of Parameters file. Note that the name of the parameter *GF_threshold* should be the same as the microenvironment entity one (e.g. *TGFBeta_threshold*). A default value can be defined with *protein_threshold*, and will be used for all densities for which this parameter was not defined by the user.

- **Glucose**: Represents glucose or nutrients needed for an active metabolism. Glucose densities in the microenvironment are calculated by the BioFVM module and can vary according to diffusions, cell uptakes/secretions and external source addition.
  Values: 1 if local density of the given glucose is high enough (above a given threshold, defined by *glucose_threshold*), 0 otherwise. If glucose is not defined in the microenvironment entities list of the Parameters file, as is the case in our cell fate model, Glucose value is set to 1 by default, thus considered not limiting, always present.
  Parameter dependency: *glucose_threshold* in cell_properties section of Parameters file (or *protein_threshold* if it is not explicitly defined).

- **Oxygen | Oxy | O2**: Represents availability of oxygen to the cell needed for an active metabolism. Oxygen densities in the microenvironment are calculated by the BioFVM module and can vary according to diffusions, cell uptakes/secretions and external source addition.
  Values: 0 if the probability of a local density of oxygen is below a given threshold: $p(O2 = 0) = r_{unfi}(0, 1)/2 < (O2_{necr} - O2_{cell})$, 1 otherwise. $O2_{cell}$ is oxygen density of oxygen in the cell's current voxel. If oxygen is not defined in the microenvironment entities list of the Parameters file, Oxygen value is set to 1 by default, thus considered not limiting, always present.
  Parameter dependency: $O2_{necr}$, threshold for oxygen level to be likely to start necrosis.

- **ECM_sensing | ECM** : Represents physical sensing of extra-cellular matrix.
  Its value will be 1 if the cell has more contacts with ECM density than a given threshold ($contact\_ecm > contact\_ecm\_threshold$, $contact\_ecm = \sum_{ecmvoxincontact:e}(cell_{rad} * (1 + f) + e_{rad} - d_{celltoe}) * e_{density})$, and 0 otherwise. f, termed *max_interaction_factor* in Parameters file, is the maximal extent of cell protrusions, meaning how far can it interact with an other cell or the matrix, as a proxy for cell protrusions such as filopodia. $e_{density}$ represents the local ECM density in the current voxel. Similar definitions are used in the case of passive cells, by looking at the overlap between passive cells and the current cell.
  Parameters dependency: *contact_ecm_threshold* and *max_interaction_factor* in cell_properties section of Parameters file as well as ECM distribution in the microenvironment as well as its density, presence/absence and voxels sizes in the ECM file.

- **DNAdamage**: Represents cell sensing DNA damage events, generally triggering Apoptosis.
  In the current version, the probability of DNA damage is more likely as nucleus "deformation" increases, as nuclear breakdown is likely to cause DNA damage (Denais *et al.*, 2016). Nucleus deformation is calculated as the overlap distance of the cell nucleus to neighbouring cells or ECM entities, divided by the nucleus radius. Values: If the nucleus deformation is greater than a given threshold ($nucleus\_deform = 0.1$ in present case, but can be changed in Parameters file), then probability of damage is $r_{unif}(0, 1) < nucleus\_deform$. As such, if the nucleus is strongly deformed (more than its radius), the probability of damage will be 1. Parameters dependency: *nucleus_deform* in cell_properties section of Parameters file.

3.2 Outputs of the Boolean model potentially considered in PhysiBoSS

- **Hypoxia**: cell will die by necrosis due to a lack of oxygen.
- **Autophagy**: cell will die by necrosis due to a lack of glucose.
- **Necrosis | NonACD**: cell commits to a necrotic process and its network is not further updated. The cell will go through a swelling phase of around 3 hours before undergoing lysis for around 45 days, as described in PhysiCell (Ghaffarizadeh *et al.*, 2018).
- **Apoptosis**: cell commits to an apoptotic process and its network is not further updated. This process includes a fast volume shrinking and cell death (cells are removed from the simulation when they reach a small size threshold) in around 8 hours (Ghaffarizadeh *et al.*, 2018).
- **Migration**: when it's ON, the amplitude of the cell's motion coefficient is increased from its current value to a maximal speed mot_{max}. When it's OFF, the coefficient is decreased until the minimal speed value mot_{min}.
  Parameter dependency: mot_{max} and mot_{min}, minimal and maximum values of the self-propelled motility amplitude.
- **Polarization**: changes the value of the current level of cell polarization, p_c, between 0 (not polarized) and 1 (fully polarized). In present case, upper limit of p_c has been limited to 0.9 to reduce the noise. When it is ON, p_c increases; when it is OFF, it decreases.
- **Cell_cell**: changes the value of the current level of the cell adhesion to other cells, p_{adh}, representing the amount of recruitment of cadherins. When it is ON, p_{adh} increases; when it is OFF, it decreases.
  Parameter: p_{adh}, and $cca_i^j(min)$, $cca_i^j(max)$.
- **Matrix_adhesion**: changes the value of the current level of cell adhesion to the matrix, p_{ecm}, representing the amount of recruitment of integrins. When it is ON, p_{ecm} increases; when it is OFF, it decreases.
  Parameter: p_{ecm}, and $cea_i(min)$, $cea_i(max)$.
- **Cell_freeze**: when ON, the cell is locked in place. The cell cannot change its volume nor actively move any more, but can be pushed or pulled. This correspond to a cell in a very crowded environment. The cell can only get out of this state unless its signalling network or environment drastically changes.
- **Quiescence**: when ON, the cell is a quiescent state. The cell is in a passive states and does not change its volume, does not proliferate, move, commit to apoptosis or die. It can however, be moved or dragged by other cells.
- **Proliferation | Survival**: if ON, the cell will progress in its cell cycle, corresponding to a Ki67-positive marker. If OFF the cell is in a quiescent phase (corresponding to a Ki67-negative marker (Ghaffarizadeh *et al.*, 2018)), unless some other output, such as Apoptosis, has been activated.
- **NFκB**: if ON, the cell will secrete TNF-α at a given rate in its surrounding matrix. If OFF, there will be no secretion.
  Parameter: rate of secretion when ON.
- **Matrix_modif**: if ON, the cell degrades the adjacent matrix at a given rate (`cell_degradation`), depending on the ECM representation chosen.
  Parameter: *mmp* rate of ECM degradation when ON.

3.3 Network state

The initial value of all the nodes of the network are defined in the MaBoSS input files. Only initial input nodes' value can be modified by the agent-based part. During the simulation, the environmental part and the Boolean model part are iteratively updated and the previous state of a cell's signalling network is reloaded at each new update. The frequency of the Boolean network update within PhysiBoSS is defined by Δt_{BN}. When a cell divides, the two daughter cells inherit the mother's network configuration and current node states, so that the same pathways are activated in the daughter cells than they were in the mother cell. When a cell commits to a state that is considered as irreversible (e.g. Apoptosis or Necrosis), its network will not be updated any more. This can be easily modified in the source code if necessary.

3.4 Time scales

Because the system involves a broad range of events at different biological scales, different time scales need to be considered. In particular, reaction-diffusion of biochemical densities in the microenvironment occurs at a very small time scale compared to cell movement, cells gaining volume or cell division (main text, Figure 1). To take these differences into account and avoid unnecessary computation of all time scales at all time steps, PhysiBoSS uses PhysiCell's three time scales (Δt_{diff} for diffusion, around 0.01 min, Δt_{mech} for cell mechanics, around 0.1 min and Δt_{cells} for cell processes, around 6 min (Ghaffarizadeh *et al.*, 2018)) and adds a fourth one: Δt_{BN} ($\Delta t_{BN} \geq \Delta t_{cells}$, around 10 min) that determines when the intracellular Boolean model is updated.

The frequency of the model update Δt_{BN} and the length of MaBoSS running time at each update t_{maxBN} are parameters that need to be carefully studied and set in a case-specific way. It is possible to model the network until it reaches its stationary behaviour, or for a shorter amount of time to take into account transient states (Stoll *et al.*, 2012, 2017). Thus, the MaBoSS evaluation time t_{maxBN} depends on the biological question of interest and the necessity or not to look at transient events.

When evaluating Boolean networks in MaBoSS, these can reach stable states given enough time receiving a constant input (Stoll *et al.*, 2012, 2017). Usually, this time to reach the stable state is model-specific and has a high variability among models. However, it is frequent in Biology that a cell will receive pulses of external signals, as for example bursts releases of TNF by immune cells and, given this dynamism, when modelling this phenomena, the signalling inside an individual cell will often be perturbed before reaching any stable state. Therefore, as we considered important to look at transient events in the signalling cascade, we chose to update the Boolean network for a given amount of time, t_{maxBN}, small enough not to reach a stable state at each run, but still long enough to avoid too frequent calls to MaBoSS engine and to allow evolution toward a new stable state. As it can be understood, the frequency of the network update within PhysiBoSS, Δt_{BN}, has to be finely tuned to the model-specific t_{maxBN}. On one hand, if the frequency of the network update within PhysiBoSS is much larger than the time of Boolean network update ($\Delta t_{BN} \gg t_{maxBN}$), the outputs of the Boolean models will not have a realistic effect on the cells' behaviour on its microenvironment; on the other hand, if the frequency is much smaller than the time of Boolean network update ($\Delta t_{BN} \ll t_{maxBN}$), the cell's physical interactions will not have effect on the behaviour of the Boolean model.

In this study, we considered that one unit of time in MaBoSS corresponded to 10 min in PhysiBoSS time. This was decided so that cell decision in response to TNF injection would occur within 1 hour of the injection, as observed experimentally. As TNF injection duration was in the order of minutes, it was important to have an update time step in the same range that would allow for sensitive input of the network. Thus, we fixed Δt_{BN} value to 10 min, which implied to take a t_{maxBN}value of 1.

References

Albert, P. J. and Schwarz, U. S. (2016). Dynamics of Cell Ensembles on Adhesive Micropatterns: Bridging the Gap between Single Cell Spreading and Collective Cell Migration. *PLoS Computational Biology*, **12**(4).

Basan, M., Elgeti, J., Hannezo, E., Rappel, W.-J. W.-J., and Levine, H. (2013). Alignment of cellular motility forces with tissue flow as a mechanism for efficient wound healing. *Proceedings of the National Academy of Sciences of the United States of America*, **110**(7), 2452–9.

Camley, B. A. and Rappel, W. J. (2014). Velocity alignment leads to high persistence in confined cells. *Physical Review E - Statistical, Nonlinear, and Soft Matter Physics*, **89**(6), 2–7.

Chu, Y.-S., Thomas, W. A., Eder, O., Pincet, F., Perez, E., Thiery, J. P., and Dufour, S. (2004). Force measurements in E-cadherin-mediated cell doublets reveal rapid adhesion strengthened by actin cytoskeleton remodeling through Rac and Cdc42. *The Journal of cell biology*, **167**(6), 1183–94.

Denais, C. M., Gilbert, R. M., Isermann, P., McGregor, A. L., te Lindert, M., Weigelin, B., Davidson, P. M., Friedl, P., Wolf, K., and Lammerding, J. (2016). Nuclear envelope rupture and repair during cancer cell migration. *Science*, **352**(6283), 353–358.

Etienne-Manneville, S. (2008). Polarity proteins in migration and invasion. *Oncogene*, **27**(55), 6970–6980.

Gerlee, P., Basanta, D., and Anderson, A. R. A. (2017). The Influence of Cellular Characteristics on the Evolution of Shape Homeostasis. *Artificial life*, **23**(3), 424–448.

Ghaffarizadeh, A., Heiland, R., Friedman, S. H., Mumenthaler, S. M., and Macklin, P. (2018). Physicell: an open source physics-based cell simulator for 3-d multicellular systems. *PLOS Computational Biology*, **14**(2), 1–31.

Haeger, A., Krause, M., Wolf, K., and Friedl, P. (2014). Cell jamming: Collective invasion of mesenchymal tumor cells imposed by tissue confinement. *Biochimica et Biophysica Acta - General Subjects*, **1840**(8), 2386–2395.

Hakim, V. and Silberzan, P. (2017). Collective cell migration: a physics perspective. *Reports on Progress in Physics*, **80**(7), 076601.

Labernadie, A., Kato, T., Brugués, A., Serra-Picamal, X., Derzsi, S., Arwert, E., Weston, A., González-Tarragó, V., Elosegui-Artola, A., Albertazzi, L., Alcaraz, J., Roca-Cusachs, P., Sahai, E., and Trepat, X. (2017). A mechanically active heterotypic E-cadherin/N-cadherin adhesion enables fibroblasts to drive cancer cell invasion. *Nature Cell Biology*, (November 2016).

Macklin, P., Edgerton, M. E., Thompson, A. M., and Cristini, V. (2012). Patient-calibrated agent-based modelling of ductal carcinoma in situ (DCIS): From microscopic measurements to macroscopic predictions of clinical progression. *Journal of Theoretical Biology*, **301**, 122–140.

Nedelec, F. (2017). preconfig : A Versatile Configuration File Generator for Varying Parameters. pages 1–4.

Stoll, G., Viara, E., Barillot, E., and Calzone, L. (2012). Continuous time boolean modeling for biological signaling: application of Gillespie algorithm. *BMC Systems Biology*, **6**(1), 116.

Stoll, G., Caron, B., Viara, E., Dugourd, A., Zinovyev, A., Naldi, A., Kroemer, G., Barillot, E., and Calzone, L. (2017). MaBoSS 2.0: an environment for stochastic boolean modeling. *Bioinformatics*, **33**(14), 2226–2228.

Szabó, A., Ünnep, R., Méhes, E., Twal, W. O., Argraves, W. S., Cao, Y., and Czirók, A. (2010). Collective cell motion in endothelial monolayers. *Physical Biology*, **7**(4), 046007.

Takeichi, M. (1991). Cadherin cell adhesion receptors as a morphogenetic regulator. *Science (New York, N.Y.)*, **251**(1989), 1451–1455.

Théry, M. and Bornens, M. (2006). Cell shape and cell division. *Current Opinion in Cell Biology*, **18**(6), 648–657.
