# Supplementary material for: PhysiBoSS: a multi-scale agent-based modelling framework integrating physical dimension and cell signalling
Source: Bioinformatics. 2018 Aug 30;35(7):1188–96. doi: 10.1093/bioinformatics/bty766 (PMC6449758; doi:10.1093/bioinformatics/bty766)
Supplement: Supplementary File S2 [file bty766_supplementary_file_s2.pdf]

Supplementary File 2: Simulations of cell fate model

Supplementary for PhysiBoSS: a multi-scale agent based modelling framework integrating physical dimension and cell signalling

Gaelle Letort, Arnau Montagud, Gautier Stoll, Randy Heiland, Emmanuel Barillot, Paul Macklin, Andrei Zinovyev, Laurence Calzone

Contents

1 MaBoSS intracellular simulations **2**
1.1 Boolean network description 2
1.2 Network initialization 2
1.3 Boolean network results 2
1.4 Mutant analyses 3

2 PhysiBoSS cell population simulations **5**
2.1 TNF/TNF-α simulation 5
2.2 Parameter table 6
2.3 Analysis of cell fate simulations 7
2.4 Supplementary study of response to TNF treatment of heterogeneous multi-cellular spheroids 8
2.4.1 Impact of resource competition in population behaviour 9

3 Annexe: MaBoSS time trajectories for WT and mutant models **13**

1 MaBoSS intracellular simulations

MaBoSS (Stoll *et al.*, 2012, 2017) is a C++ software for stochastically simulating continuous/discrete time Markov processes defined on the state transition graph describing the dynamics of a logical model. In MaBoSS framework, the rates up (change from OFF to ON) and down (from ON to OFF) for each variable of the model can be explicitly defined to represent physical kinetic rates of the variables' turnover (Stoll *et al.*, 2012). Probabilities to reach a phenotype are thus computed by simulating random walks on the probabilistic state transition graph.

1.1 Boolean network description

One MaBoSS model consists in a network defining the logical relations between all the nodes (gene) in the pathways of interest. Its structure (which gene activates/inhibits other genes) is built from literature sources. In this study, the Boolean model focuses on pathways leading to cellular fates (Fig SI 1). It was adapted from Calzone *et al.* (2010) and comprises 31 nodes and 50 edges.

Inputs of the model are TNF, FADD and FASL, whose presence activates different highly intertwined pathways leading to *Survival*, *Apoptosis* or non-apoptotic cell death (*NonACD*, which mainly covers necrosis). A caspase-dependent apoptotic cell death pathway has been considered by including the caspase-dependent pathway downstream of FASL and TNF receptors, with the presence of FADD. A mode of cell death with morphological features of necrosis has been considered, which occurs when apoptosis is impeded in cells treated with cytokines (Holler *et al.*, 2000) or in some specific cell lines such as L929 cells when exposed to TNF (Fiers *et al.*, 1995). Additionally, a family of transcription factors around NFκB that play a central role in inflammation, immune response to infections and cancer development have been included in the model to represent a survival pathway by means of inhibiting TNF-promoted apoptosis engagement. The construction of the model has been described in detail elsewhere (Calzone *et al.*, 2010).

It is important to note that not much is known about timing in *Apoptosis* and *NonACD* commitment to this cell fate. In the construction of the network, we chose to have a possible activation of *Apoptosis* much faster than *NonACD* activation. Indeed, *NonACD* corresponds to both necrosis (when ATP drops dramatically as a result of, for instance, a compromised mitochondrial membrane) and necroptosis (when necrosis is programmed via gene RIP1), and *in vitro* it has been shown that necroptosis occurs when apoptosis is compromised. With this model, the activity of mXIAP had a strong impact on cell fate decision: when easily activated (e.g. if transcription rate is low), it will activate easily XIAP, which will inhibit the apoptosome and CASP3 (Fig SI 1), favouring *NonACD* fate.

1.2 Network initialization

In all our simulations, cells were initially proliferating. To simulate healthy functioning cells, all internal nodes were initially set to 0, except ATP and cIAP which were set to 1 (Calzone *et al.*, 2010). Regarding inputs, FASL was set to 0 and FADD value was randomly chosen (0 or 1) for each cell; TNF value varied during the simulations according to amount of TNF-α bound to each cell (initially 0).

1.3 Boolean network results

Results for MaBoSS can be visualised in two manners: the trajectories for particular model states can be interpreted as the evolution of a cell population as a function of time; that way, transient effects could be highlighted. Alternatively, asymptotic solutions can be represented as pie charts to illustrate the proportions of cells in particular model states. This second

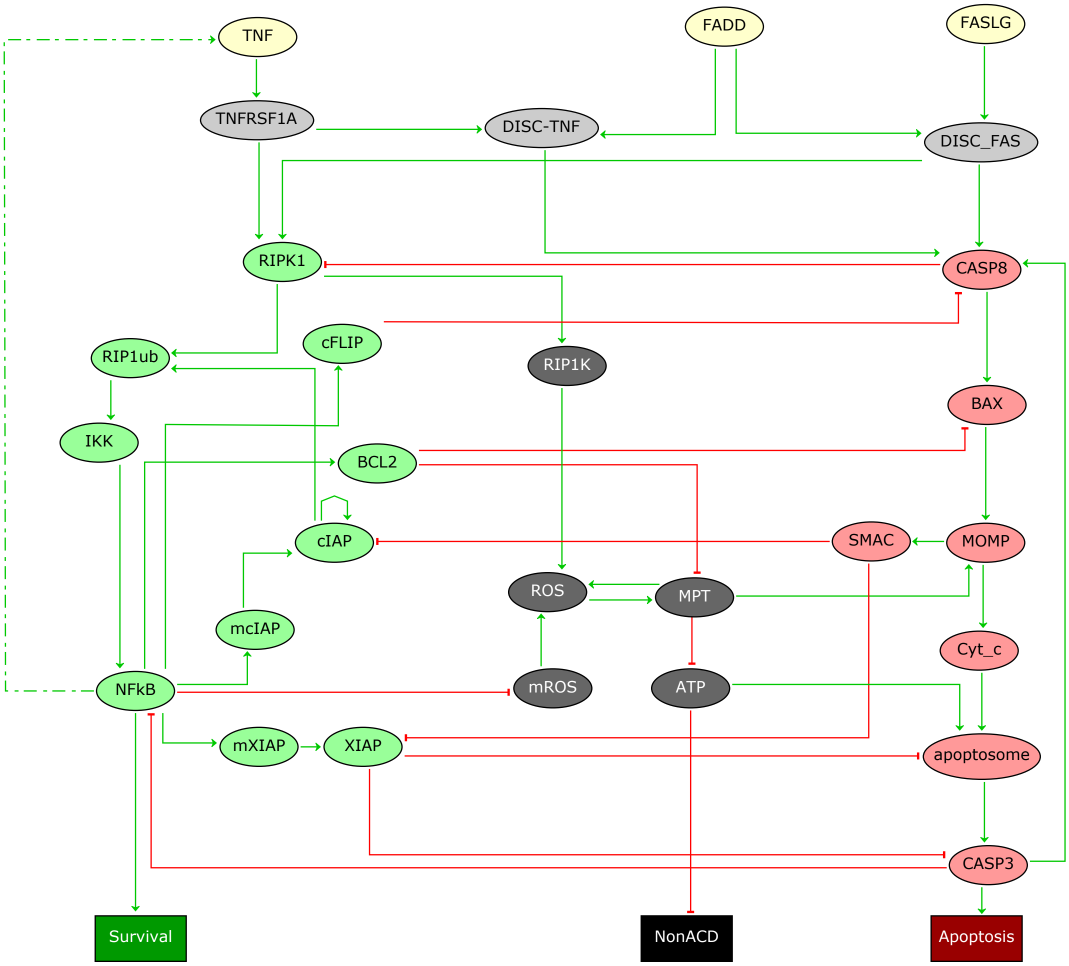

Fig 1. Cellfate model Boolean network adapted from Calzone *et al.* (2010). Inputs are yellow, *Survival* pathway is green, *Necrosis* pathway is black and *Apoptosis* pathway is red. Nodes starting by lowercase "m" represent mRNAs. Dashed line represents NFκB to TNF feedback loop and is not present explicitly in the Boolean model, but in the agent-based part of PhysiBoSS.

representation is useful when comparing two conditions (e.g. altered environment conditions or component perturbations) where the proportions of the model states change from one condition to another. For instance, in MaBoSS simulation of the WT model with TNF presence, these initial conditions resulted in a population of cells were 48.4% of the cells committed to *Apoptosis*, 46.2% committed to *Survival* and 5.4% had been committed to *NonACD* (Fig SI 2A).

1.4 Mutant analyses

We called mutant a strain that has a Boolean network modified (mutated) from the default (wild-type) one, by differences in transition rates. We used only "extreme" mutations: to simulate a gene over-expression, its ON rate was set to a very high value, and its OFF rate was set to 0. Similarly, knock-down of a gene was mimicked by an ON rate of 0 and very high OFF rate.

We begun by selecting interesting mutations with MaBoSS simulations before testing them in PhysiBoSS. A previously published pipeline on computational tools for logical models has been used to explore mutants of this model (Montagud *et al.*, 2017). Mutants that maximized

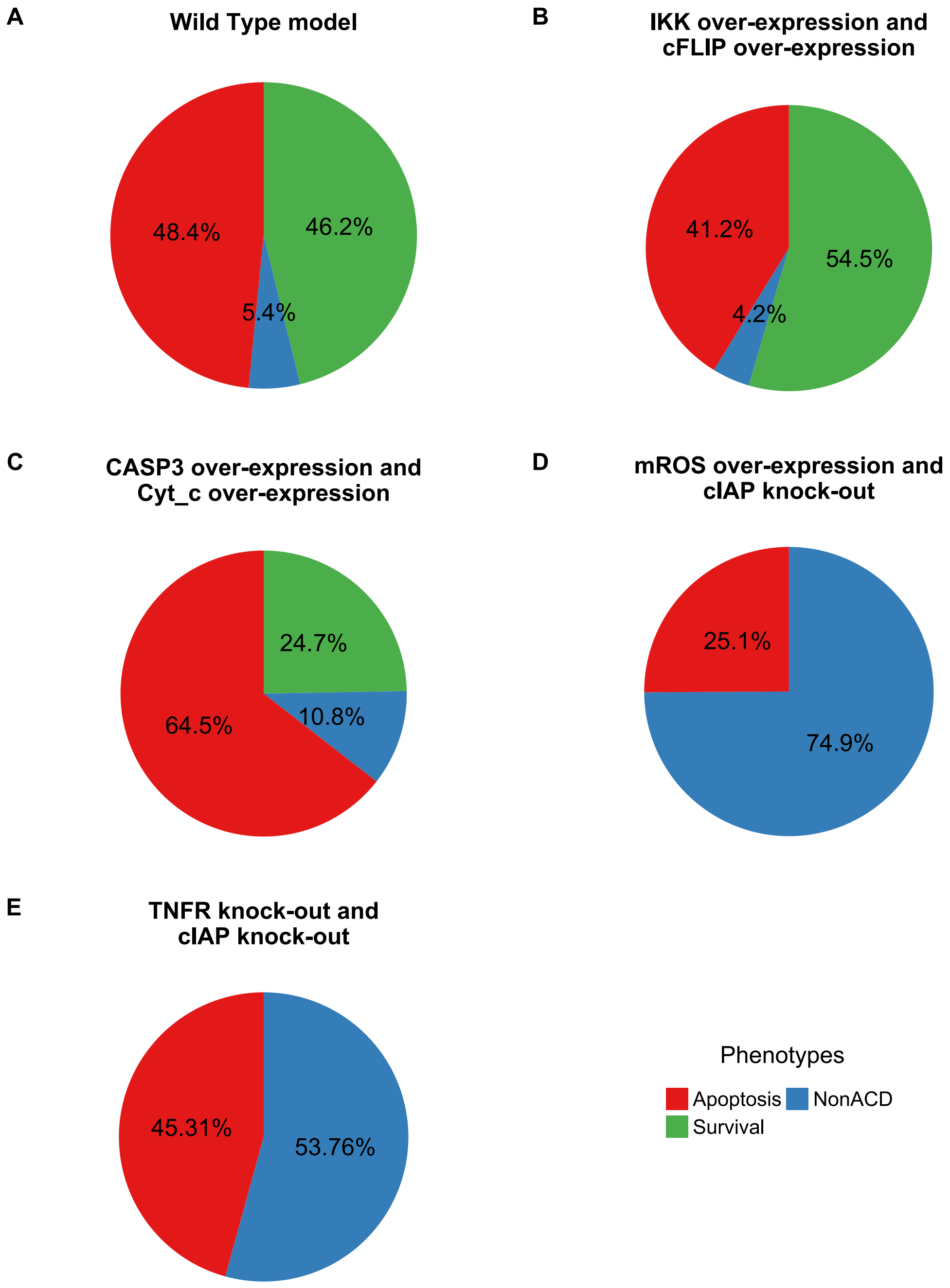

Fig 2. MaBoSS results for wild type and mutant models. A, Wild Type model; B, Mutant with over-expressed IKK and cFLIP; C, Mutant with over-expressed CASP3 and Cyt_c; D, Mutant with over-expression of mROS and knock-out of cIAP; E, Mutant with knocked-out of TNFR and cIAP.

phenotype changes in respect to WT phenotype proportions were selected. Following the instructions of the pipeline, all possible single and double mutants were simulated (totalling 1569 models) and their results were analysed.

The double mutant of IKK and cFLIP over-expression was selected for its increased *Survival* phenotype (Fig SI 2B). The double mutant of CASP3 and Cyt_c over-expression was selected as it had an increased *Apoptosis* phenotype and a diminished Survival one (Fig SI 2C). Also, the double mutant of mROS over-expression and cIAP knock-out was selected as it had an increased *NonACD* phenotype as well as diminished *Apoptosis* and *Survival* phenotypes (Fig SI 2D). The different proportions can be studied in Figure 2 and the time trajectories of the WT and mutants models can be found at the end of this file.

2 PhysiBoSS cell population simulations

PhysiBoSS is available on GitHub (https://github.com/sysbio-curie/PhysiBoSS) and is presented in the main text. Here, we precise more informations on the simulations presented in this paper.

At the initial time of the simulation, cells were proliferative, and placed randomly in a given geometry (a sphere of radius 100 μm in these examples), with a cell radius uniformly chosen (between the input parameter radius r and $\sqrt[3]{2}r$). Except for the cell sorting simulations, we always used the same cells parameters that are given in the parameter table further below.

2.1 TNF/TNF-α simulation

To simulate the synergy between injected TNF-α in the medium and cell's signalling pathway activated by its internalization, we simulated TNF as a density present in the extra-cellular matrix that can be consumed by cells from the first time point. TNF dynamics are tricky to implement as, even though some parameters can be assessed from the literature (e.g. TNF-α diffusion, decay rate...), others are difficult to measure experimentally, such as binding of TNF to cells and internalization and secretion rates, and thus are poorly documented.

Here, we simulated TNF-α binding to the cell receptors by retrieving a given amount of TNF-α from the cell-neighbouring environment and adding it to a cell's internal accumulator. Diffusion, natural degradation, cell uptake and secretion are handled by the BioFVM module (Ghaffarizadeh *et al.*, 2016). We added the cell accumulator to keep track of how much TNF was consumed (bound to the cell). 1 % of those bounded TNF was degraded spontaneously every minute, so if no additional TNF was provided to the media, it would decrease and disappear after some time. When the amount of bounded TNF reached a given threshold, we considered it to be internalized by the cell and it activated the corresponding pathway (TNF input of the Boolean network is set to 1). This Boolean input node will be de-activated (set to 0) as soon as the level of bound TNF decreases below the given threshold. All these parameters are detailed in the table below.

Injection of TNF in the medium was simulated in two different modes: uniformly adding it in all the medium (microfluidic-like) or at the periphery of the simulation space only (local injection). In the first mode, all voxels of the simulation space are set to have a given concentration. This can be used to represent a microfluidic system where all the medium is replaced by the injected one nearly instantaneously. At the end of the injection (if this is not continuous), the chamber is washed-off (replaced by a neutral solution). This was simulated by removing all TNF from the simulation space (note however that TNF can nonetheless appear in the medium afterwards as it can be secreted by the cells).

In the second mode, only voxels at the periphery of the simulation space are set to bear the injected concentration, mimicking an external contribution of TNF solution, during all the time of the injection. In this case, TNF is not washed-off at the end of the injection. This is the method used in our spheroid assays. When oxygen diffusion was also simulated, we considered it to be provided during all the simulation from the exterior boundaries of the simulation space, thus using this second mode.

2.2 Parameter table

A list of the main parameters used in the simulation presented here is given in the table below. Complete parameter files are provided in PhysiBoSS repository.

Name	Value	Details
**Simulation**		
Time step	0.02 min	Diffusion time scale, termed Δt_{diff} (Ghaffarizadeh *et al.*, 2018)
Mechanical step	0.1 min	Cell volume gain, motion time scale, termed Δt_{mech} (Ghaffarizadeh *et al.*, 2018)
Cycle time step	2 min	Cell cycle time scale, change of phase, termed Δt_{cells} (Ghaffarizadeh *et al.*, 2018)
Voxel size	15 μm^3	Spatial discretisation of the simulated space
**Cell strain**		
Adhesion strength	2	Empirical, a value of 2 means a strong adhesion between cells. Here we used the same value for homotypic and heterotypic adhesions
Motility amplitude	0.01	Empirical, determines how much cells can move, a value of 0.01 is very low in this case
Cell-cell repulsion	10	Coefficient of repulsion when cells overlap
Cell radius	8.5 μm	Cell radius just after division, approximate value for 3T3 fibroblast cells
Cell cycle time	16 h	Doubling time of 3T3 cells (Kim *et al.*, 2004)
Apoptosis time	5-8 h	Duration of apoptosis (Ghaffarizadeh *et al.*, 2018)
**TNF simulation**		
TNF diffusion	$2.10^{-7} cm^2/s$	Diffusion speed of free TNF-α in the medium (Cheong *et al.*, 2006)
TNF decay rate	0.03 min^{-1}	Due to natural degradation and adherence to ECM proteins, half-life of 25 min (Cheong *et al.*, 2006)
Uptake rate	0.0025 $voxel^{-1}.min^{-1}$	Quantity of TNF consumed by a cell, empirical
Internalisation threshold	$2.8\,10^{-5}$	Quantity of accumulated TNF in a cell's surroundings to consider it as internalised, empirical
Secretion rate	0.1 fg/cell/min	Speed of TNF production by a cell when NFκB is ON. Value was chosen in the range of plausible secretion rate from Liu *et al.* (2012).
**Oxygen dynamics**		
Oxygen concentration	10	Oxygen input concentration per voxel, empirical
Necrotic threshold	8.5	Oxygen level below which necrosis will appear

Oxygen diffusion	$1.10^5 \mu m^2/min$	oxygen diffusion speed (Ghaffarizadeh *et al.*, 2016)
**Boolean network**		
Update time step	10 min	Frequency of network updating, termed Δt_{BN}
Maximal time	1	maximal time iteration in MaBoSS pseudo-time, termed t_{maxBN}. A MaBoSS time of 1 thus corresponds to 10 min in PhysiBoSS.
Number of trajectories	1	Number of times the Gillespie algorithm was run on the state transition graph per cell. Here we have used one trajectory per cell.
MaBoSS time step	0.01	Time step in MaBoSS evaluation. Each time the network is updated, the maximal time is divided by these time steps. For a thorough discussion on the interplay of these time steps and the maximal time, please refer to Stoll *et al.* (2012).
Transition rate	1	Default transition rate of nodes in MaBoSS.
Transcription rate	1/24	Transition rate of nodes in MaBoSS representing transcription events, slower than other nodes that are meant to represent protein-protein interaction or signalling cascades.

The complete list of the parameters, and their range of values advised are given in the GitHub repository (https://github.com/sysbio-curie/PhysiBoSS/wiki/Parameters). In the table above, we provided the values that corresponded to our present study only. To illustrate the effect of choosing different values for a given parameter in our study, we varied the concentration of TNF in the case of a continuous injection (WT cells spheroid), the amount of TNF intake by a cell, and numerically-related parameters such as the frequency of MaBoSS evaluation or the running time of one MaBoSS call (Fig 3).

2.3 Analysis of cell fate simulations

For each configuration, we simulated 5 independent simulations with the exact same parameter sets. In PhysiBoSS, stochasticity is introduced at different levels (on initial configuration, cell cycle timing, Boolean network transitions, etc.), but its effect is small compared to the deterministic clues. Thus, simulating 5 times by condition was representative enough.

To measure the fraction of cells that are activated in response to TNF and compare it with the results from the experimental studies (Kellogg *et al.*, 2015; Tay *et al.*, 2010), we kept track of NFκB activation in the Boolean network. As soon as this node got activated once, even if it didn't stay activated, we considered the cell as activated, following the "activated" definition of Tay *et al.* (2010) and Kellogg *et al.* (2015). This corresponds to the experimental observation of oscillatory NFκB translocation in the nucleus in response to TNF injection. We then compared the number of cells that got activated to the initial total number of cells in the simulation set-up.

Analyses of the simulations were performed in Python, and the scripts are available in the GitHub repository (https://github.com/sysbio-curie/PhysiBoSS/tree/master/scripts). Figures of this publication were created with Paraview and Inkscape.

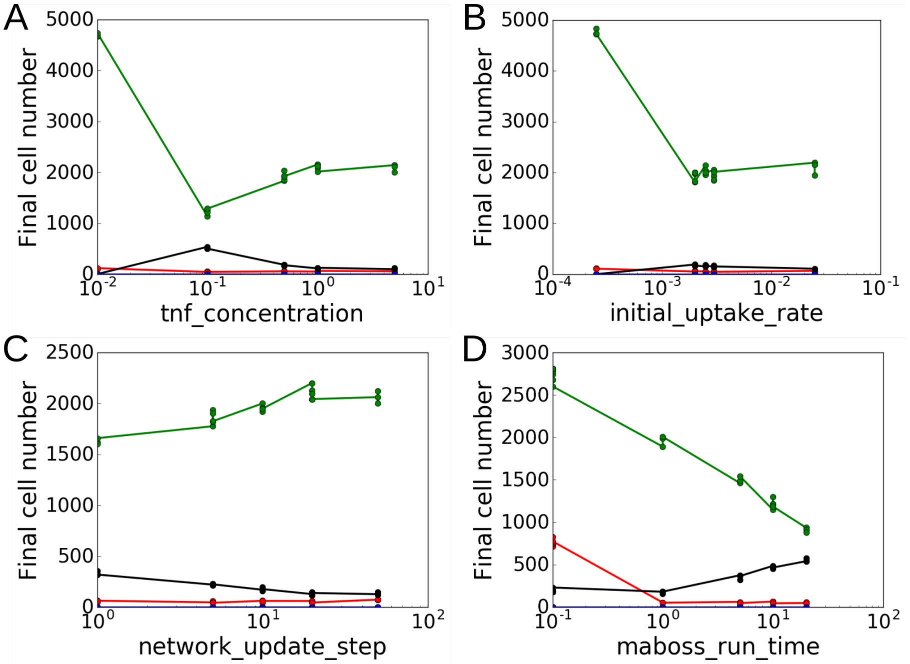

Fig 3. Example of parameter values variation. A: Final number of cells committed to each fate according to the continuously injected TNF concentration value. B: Final number of cells committed to each fate according to the TNF intake value of each cell. C: Final number of cells committed to each fate for different update frequency of the MaBoSS network (Δt_{BN}). D: Final number of cells committed to each fate for different length of MaBoSS running at each call (t_{maxBN}). A-D: Colour code: green, *Proliferative* cells; red, cells committed to *Apoptosis*; black, cells committed to *NonACD*. Initial spheroid radius is 100 μm, which accounts for roughly 1000 cells.

2.4 Supplementary study of response to TNF treatment of heterogeneous multi-cellular spheroids

One major challenge in tumour treatment is the high level of heterogeneity in the population. In a tumour environment, different cells with different mutations are present and competing for resources such as growth factors, nutrients and space. To handle such complex system, a tool like PhysiBoSS is very useful as it can simulate and study the evolution of clones in the population.

To illustrate this, in previous section (Mutant analyses) using MaBoSS framework, we identified interesting mutation phenotypes on our network. These were then simulated in PhysiBoSS spheroid population with 75% of the initial population as wild type (non mutated, WT) and 25% mutated. We simulated a mutant with over-expression (+) of mROS and cIAP knock-out (-) (Fig 4A,B), which was found to favour necrosis in response to TNF treatment. Accordingly, none of the mutated cells displayed *Proliferative* state after 24 h, very few were committed to *Apoptosis* and most of them were committed to *NonACD* (Fig 4A,B). We also simulated a CASP3+ and Cytc+ double mutant (Fig 4 A,B), which was known to favour apoptosis. Indeed, in the spheroid set-up, the mutated cells underwent Apoptosis in response to TNF and only the WT population remained after 24 h. Finally, we simulated an IKK+ and cFLIP+ mutant sub-population (Fig 4A,B), that was known to favour *Proliferation* response. As expected, the mutant population survived TNF treatment and proliferated in the spheroid overtaking WT strain space as these died out. These is the mutant discussed in the main text.

In these simulations, the presence of the mutated population didn't impact much the growth of the WT population: the final ratio of surviving WT cells compared to their initial number

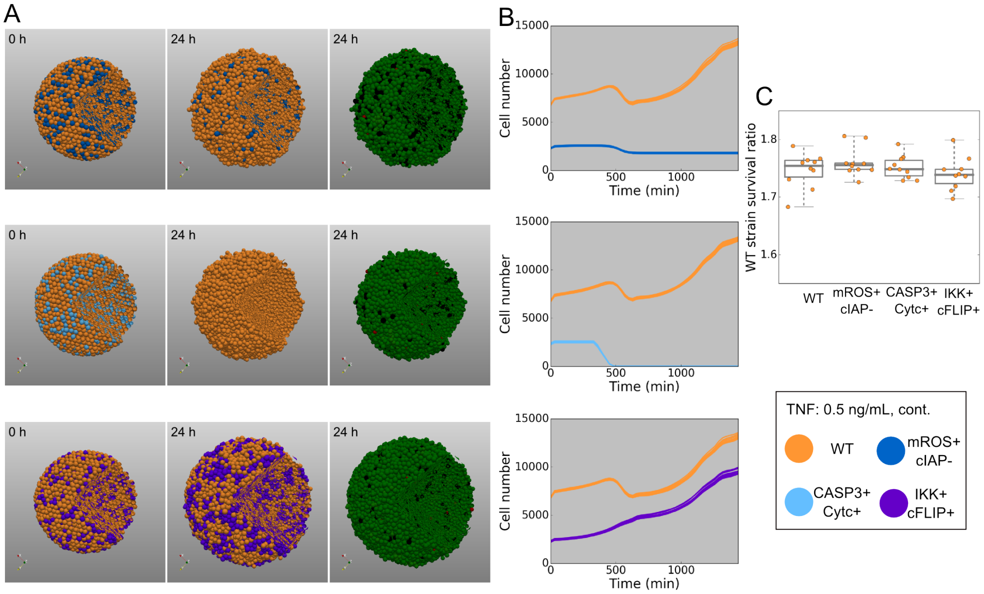

Fig 4. Genetically heterogeneous population under TNF treatment. Simulations of heterogeneous population composed of 75% of WT cells (orange) and 25% of mROS+ and cIAP- mutated cells (blue, top) or 25% of CASP3+ and Cytc+ mutated cells (light blue, middle) or 25% of IKK+ and cFLIP+ mutated cells (purple, bottom). A: Snapshots of a simulation for each case at initial and final time (24 h), with cells coloured by cell line (left and middle) or by cell fate commitment (right). B: Time evolution of the number of cells in each strain (WT and mutated) for 10 simulations. Grey shading indicates presence of TNF in continuous injection at 0.5 ng/mL. C: Ratio of final number of surviving WT cells against their initial number for each simulation. A-C: Colour code: green, *Proliferative* cells; red, cells committed to *Apoptosis*; black, cells committed to *NonACD*. Initial spheroid radius is 200 μm, which accounts for roughly 9000 cells, + stands for over-expression and - stands for knock-out.

was similar to the one in a WT-only population (Fig 4C, no significant difference under Kolmogorov-Smirnov test). This was also true when simulating different initial WT/mutant cells ratios (main text S3 FigC, 50% and 25% of WT proportion tested). Notably, PhysiBoSS can handle simulation with more than two strains. To illustrate an example of a three strain problem, namely WT, CASP8 knock-out and CASP8 over-expressed strains, please refer to our GitHub repository (https://github.com/sysbio-curie/PhysiBoSS/wiki/Example_Mutants). In fact PhysiBoSS can handle as many strains as cells in a population, if they are properly described in its parameter file, in a disk (2D) or in a sphere (3D) set-up.

2.4.1 Impact of resource competition in population behaviour

To further mimic a crowded environment such as tumours and study the impact of resource competition among cell strains for a given resource, we took advantage of the ability of PhysiBoSS to integrate environmental and internal clues. This consumable resource could be a chemical specie such as oxygen, nutrients, growth factors, etc.

To illustrate this point, we considered oxygen diffusion and its cell consumption in the 3D spheroid set-up. Oxygen was used here as a proxy for limited resources. A threshold under which cell commit to necrosis due to lack of oxygen was fixed (Parameter table). As a consequence, a necrotic core formed in the centre of the spheroid, where oxygen levels were very low, with a thin proliferative rim around it (Fig 5A), as it has been described for large spheroids (Freyer and Sutherland, 1986; Sutherland, 1988). Therefore, under TNF treatment, the population growth

was strongly limited by the combination of TNF- and oxygen-mediated death. A sub-population of cells still resisted the treatment through NFκB activation and oxygen availability (Fig 5B).

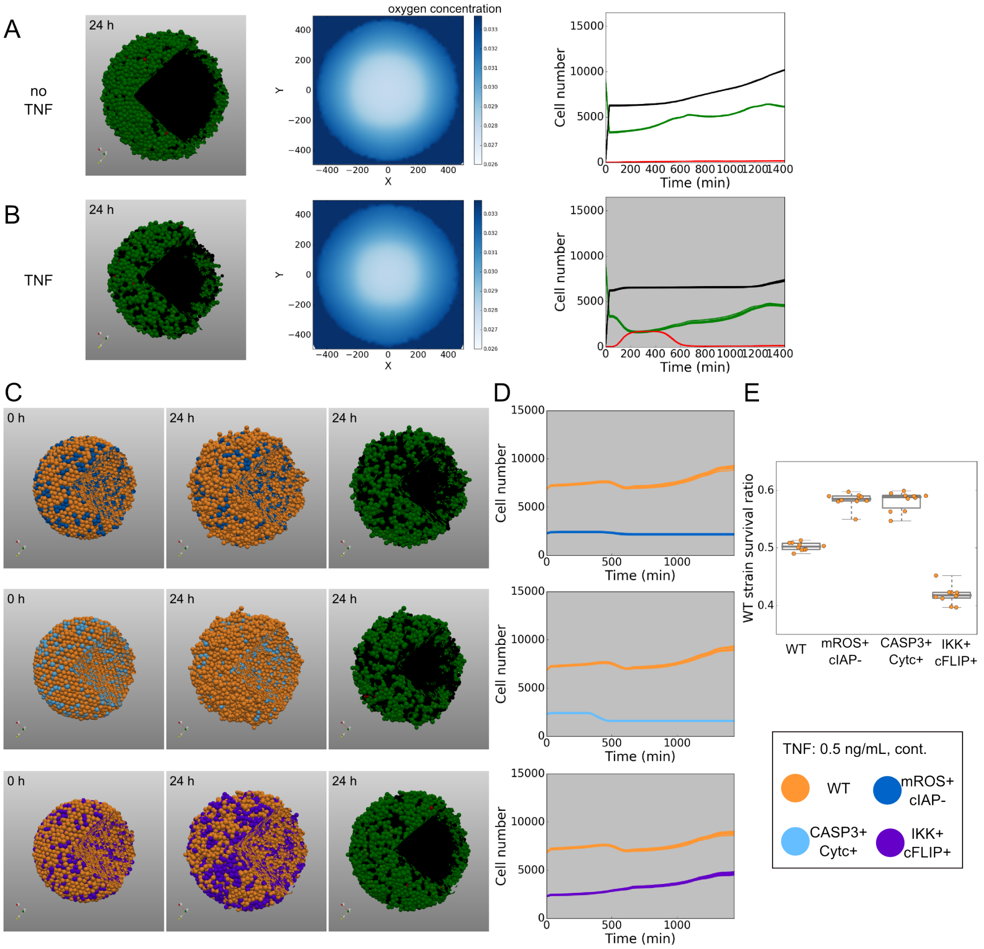

Fig 5. Spheroid response to TNF injection in oxygen-limited regime. A: Cell fates' simulation without TNF. Snapshots of a simulation (left) and oxygen levels (middle) at 24 h. Time evolution of the number of cells in each cell fate (right). B: Cell fates' simulation for a low-dose injection of TNF. Snapshots of a simulation (left) and oxygen levels (middle) at 24 h. Time evolution of the number of cells in each cell fate (right). C-E: Simulations of heterogeneous population composed of 75% of WT cells (orange) and 25% of mROS+ and cIAP- mutated cells (blue, top) or 25% of CASP3+ and Cytc+ mutants (light blue, middle) or 25% of IKK+ and cFLIP+ mutants (purple, bottom) under continuous TNF treatment. C: Snapshots of a simulation for each case at initial and final time (24 h), with cells coloured by cell line (left and middle) or by cell fate commitment. D: Time evolution of the number of cells in each strain (WT and mutated) for 10 simulations. E: Ratio of final number of surviving WT cells against their initial number for each spheroid simulation of heterogeneous population with TNF. A-E: Oxygen levels, represented by blue shades, are measured in the z = 0 plane of the simulated space. Colour code: green, *Proliferative* cells; red, cells committed to *Apoptosis*; black, cells committed to *NonACD*. Grey shading indicates presence of TNF in continuous injection at 0.5 ng/mL. All simulations are in an oxygen-limited regime, initial spheroid radius is 200 μm, which accounts for roughly 9000 cells, + stands for over-expression and - stands for knock-out.

Because of this competition for oxygen, WT population growth was affected by the response of the mutated population (Fig 5E). Indeed, the TNF-mediated death of mROS+ and cIAP-

mutant and CASP3+ and Cytc+ mutant, freed space that allowed WT cells to have an increased access to oxygen, proliferating slightly more than the homogeneous WT-only spheroid (Fig 5E). This effect was persistent even if we decreased the proportion of WT cells to 25% (S3 FigD in Main text). However, when studying the heterogeneous spheroid with WT cells and CASP3+ and Cytc+ mutants, WT strain survival rate was lower than the homogeneous WT-only spheroid (Fig 5E). This was due to CASP3+ and Cytc+ mutation favouring the survival of cells (Fig 5D), thus competing with the WT strain for oxygen and space and decreasing WT numbers, when compared to the WT-only spheroid (Fig 5E and main text, S3 FigD).

These results evidence that competition among strains cannot be neglected as the resulting tumour is consequence of the different adaptive ability of these to the environmental conditions and to the TNF treatment, that may result in the growth of resistant clones that gain access to nutrients.

References

Calzone, L., Tournier, L., Fourquet, S., Thieffry, D., Zhivotovsky, B., Barillot, E., and Zinovyev, A. (2010). Mathematical modelling of cell-fate decision in response to death receptor engagement. *PLoS Computational Biology*, **6**(3), e1000702.

Cheong, R., Bergmann, A., Werner, S. L., Regal, J., Hoffmann, A., and Levchenko, A. (2006). Transient IκB kinase activity mediates temporal NF-κB dynamics in response to a wide range of tumor necrosis factor-α doses. *Journal of Biological Chemistry*, **281**(5), 2945–2950.

Fiers, W., Beyaert, R., Boone, E., Cornelis, S., Declercq, W., Decoster, E., Denecker, G., Depuydt, B., De Valck, D., De Wilde, G., Goossens, V., Grooten, J., Haegeman, G., Heyninck, K., Penning, L., Plaisance, S., Vancompernolle, K., Van Criekinge, W., Vandenabeele, P., Vanden Berghe, W., Van de Craen, M., Vandevoorde, V., and Vercammen, D. (1995). TNF-induced intracellular signaling leading to gene induction or to cytotoxicity by necrosis or by apoptosis. *Journal of inflammation*, **47**(1-2), 67–75.

Freyer, J. P. and Sutherland, R. M. (1986). Regulation of growth saturation and development of necrosis in EMT6/Ro multicellular spheroids by the glucose and oxygen supply. *Cancer research*, **46**(7), 3504–12.

Ghaffarizadeh, A., Friedman, S. H., and Macklin, P. (2016). BioFVM: An efficient, parallelized diffusive transport solver for 3-D biological simulations. *Bioinformatics*, **32**(8), 1256–1258.

Ghaffarizadeh, A., Heiland, R., Friedman, S. H., Mumenthaler, S. M., and Macklin, P. (2018). Physicell: an open source physics-based cell simulator for 3-d multicellular systems. *PLOS Computational Biology*, **14**(2), 1–31.

Holler, N., Zaru, R., Micheau, O., Thome, M., Attinger, A., Valitutti, S., Bodmer, J.-L., Schneider, P., Seed, B., and Tschopp, J. (2000). Fas triggers an alternative, caspase-8–independent cell death pathway using the kinase RIP as effector molecule. *Nature Immunology*, **1**(6), 489–495.

Kellogg, R. A., Tian, C., Lipniacki, T., Quake, S. R., and Tay, S. (2015). Digital signaling decouples activation probability and population heterogeneity. *eLife*, **4**(OCTOBER2015), 1–26.

Kim, A., Zhong, W., and Oberley, T. D. (2004). Reversible Modulation of Cell Cycle Kinetics in NIH/3T3 Mouse Fibroblasts by Inducible Overexpression of Mitochondrial Manganese Superoxide Dismutase. *Antioxidants & Redox Signaling*, **6**(3), 489–500.

Liu, Y., Kwa, T., and Revzin, A. (2012). Simultaneous detection of cell-secreted TNF-?? and IFN-?? using micropatterned aptamer-modified electrodes. *Biomaterials*, **33**(30), 7347–7355.

Montagud, A., Traynard, P., Martignetti, L., Bonnet, E., Barillot, E., Zinovyev, A., and Calzone, L. (2017). Conceptual and computational framework for logical modelling of biological networks deregulated in diseases. *Briefings in Bioinformatics*.

Stoll, G., Viara, E., Barillot, E., and Calzone, L. (2012). Continuous time boolean modeling for biological signaling: application of Gillespie algorithm. *BMC Systems Biology*, **6**(1), 116.

Stoll, G., Caron, B., Viara, E., Dugourd, A., Zinovyev, A., Naldi, A., Kroemer, G., Barillot, E., and Calzone, L. (2017). MaBoSS 2.0: an environment for stochastic boolean modeling. *Bioinformatics*, **33**(14), 2226–2228.

Sutherland, R. M. (1988). Cell and environment interactions in tumor microregions: the multicell spheroid model. *Science (New York, N.Y.)*, **240**(4849), 177–84.

Tay, S., Hughey, J. J., Lee, T. K., Lipniacki, T., Quake, S. R., and Covert, M. W. (2010). Single-cell NF-κB dynamics reveal digital activation and analogue information processing. *Nature*, **466**(7303), 267–271.

3 Annexe: MaBoSS time trajectories for WT and mutant models

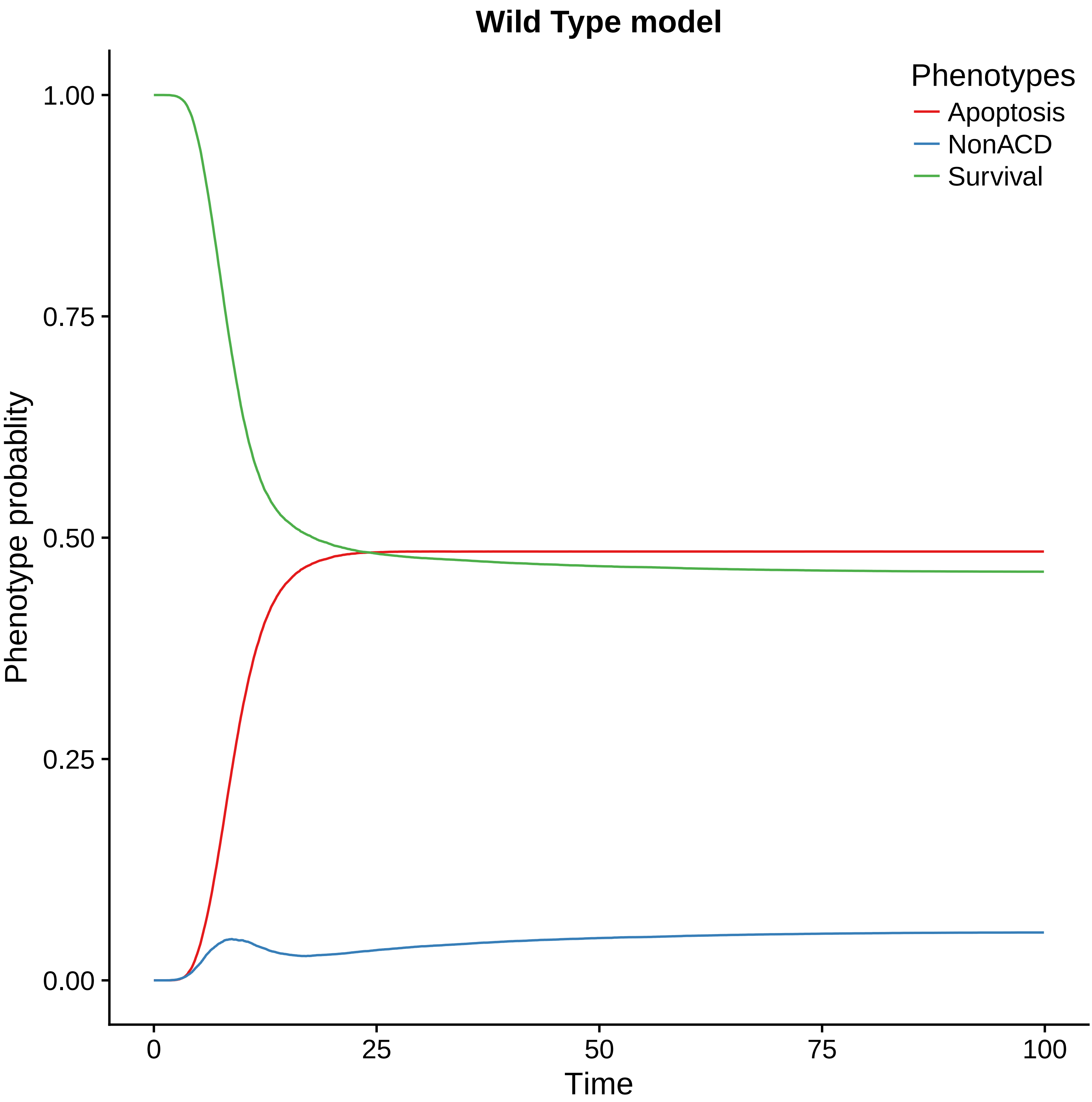

Wild Type model
Phenotypes
Apoptosis
NonACD
Survival
1.00
0.75
0.50
0.25
0.00
Phenotype probablity
0
25
50
75
100
Time

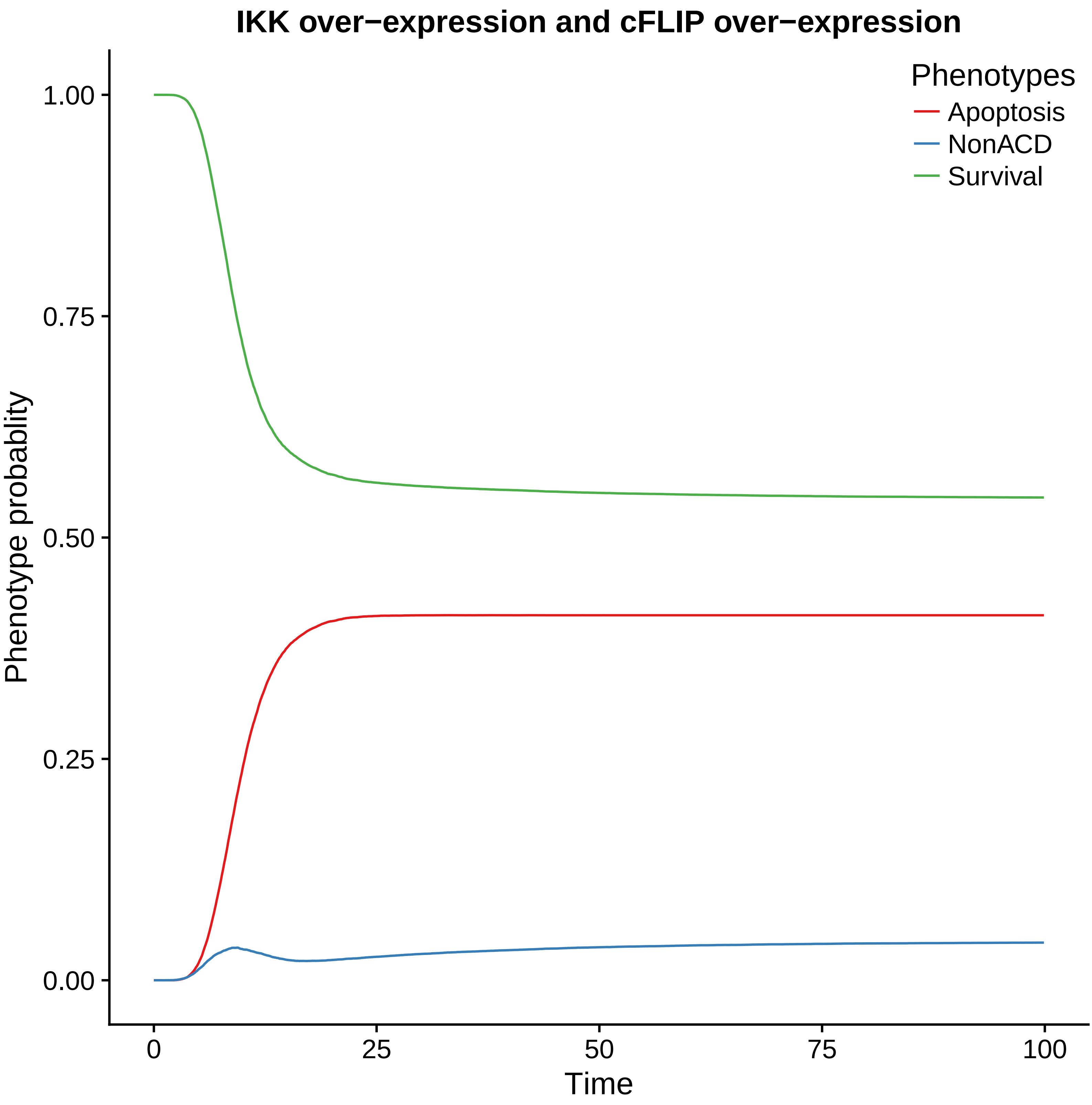
IKK over−expression and cFLIP over−expression
Phenotypes
Apoptosis
NonACD
Survival
Phenotype probablity
1.00
0.75
0.50
0.25
0.00
0
25
50
75
100
Time

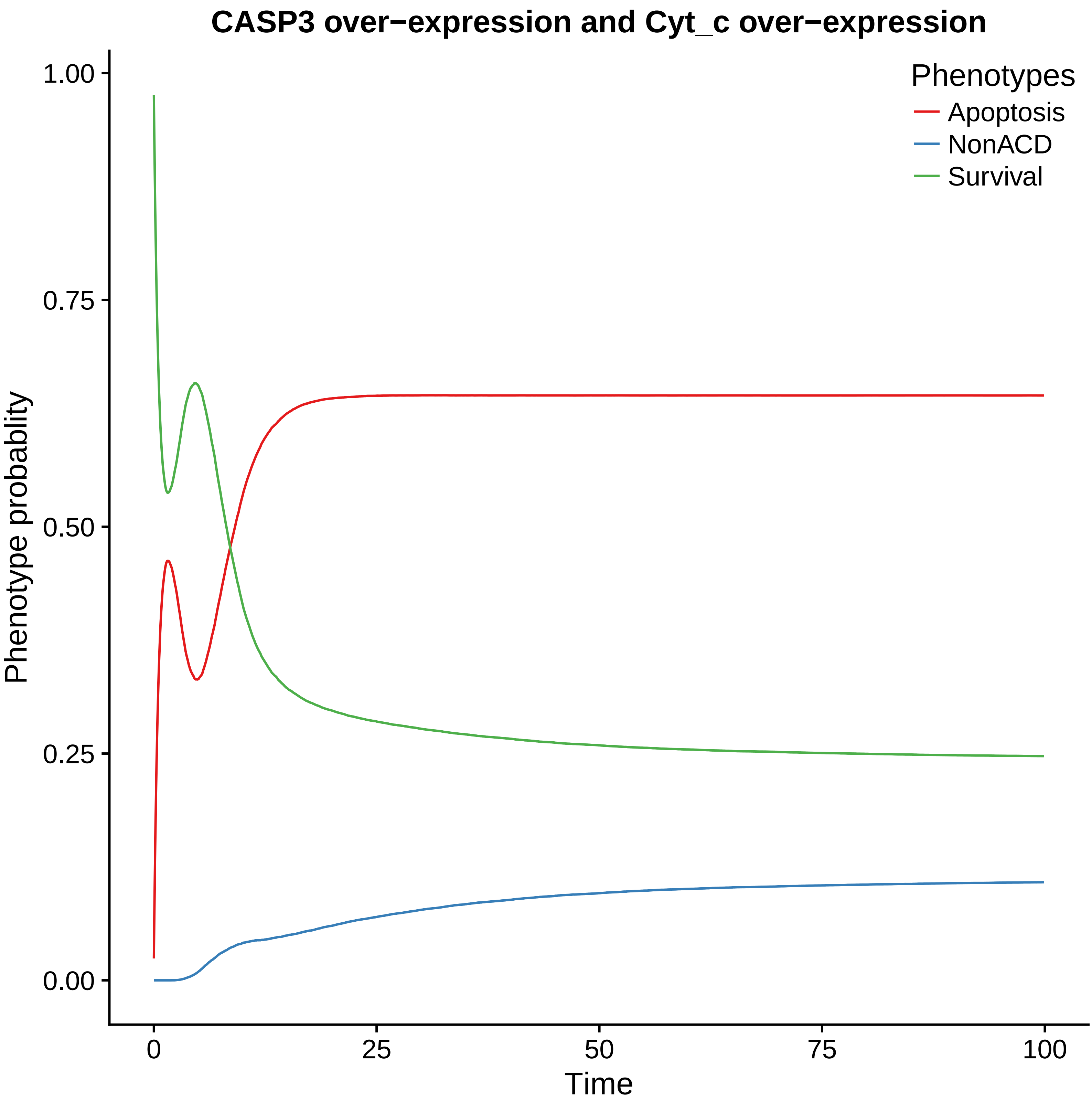
CASP3 over−expression and Cyt_c over−expression
Phenotypes
Apoptosis
NonACD
Survival
1.00
0.75
0.50
0.25
0.00
Phenotype probablity
0
25
50
75
100
Time

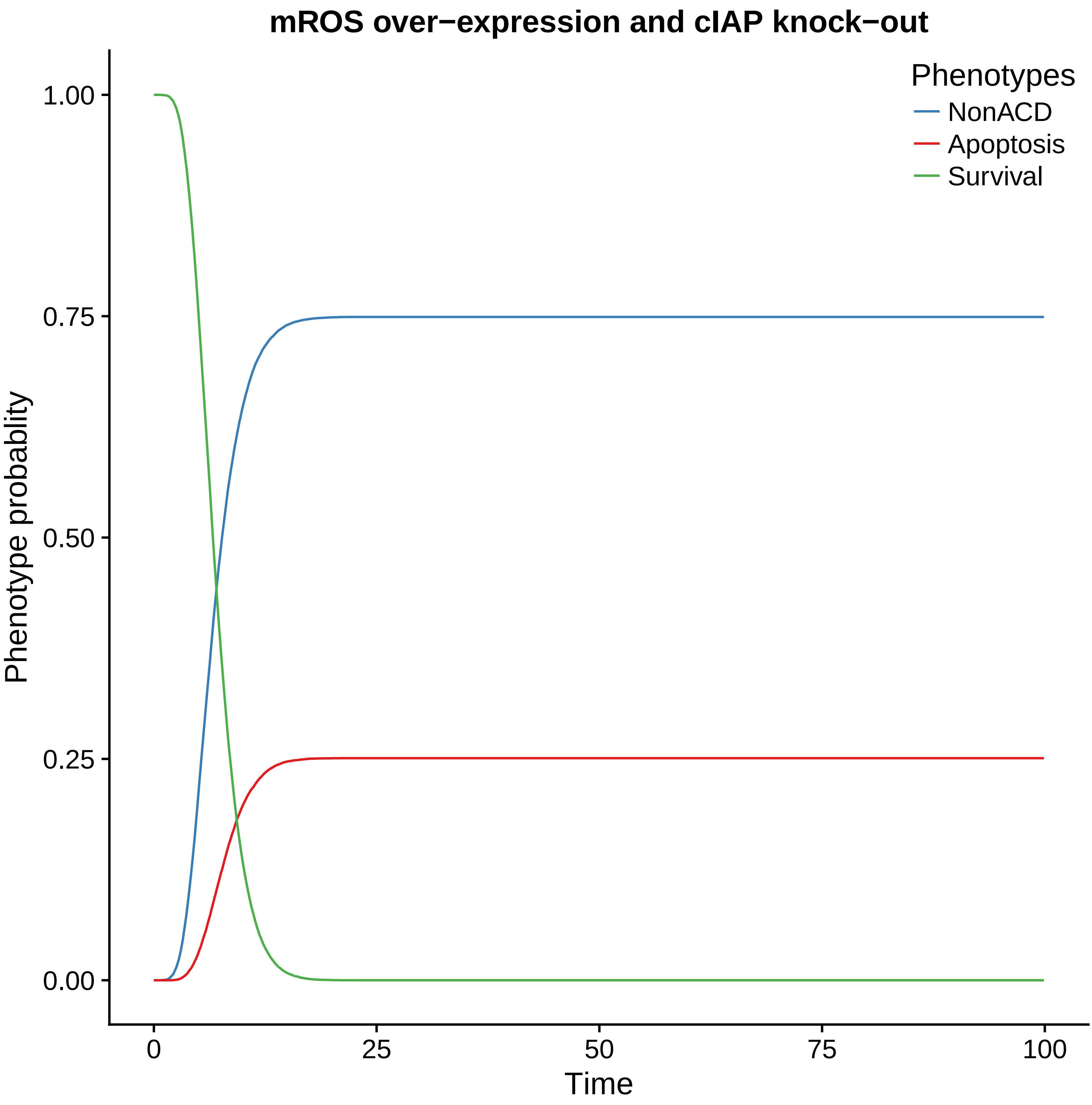
mROS over−expression and cIAP knock−out
Phenotypes
NonACD
Apoptosis
Survival
Phenotype probablity
1.00
0.75
0.50
0.25
0.00
0
25
50
75
100
Time

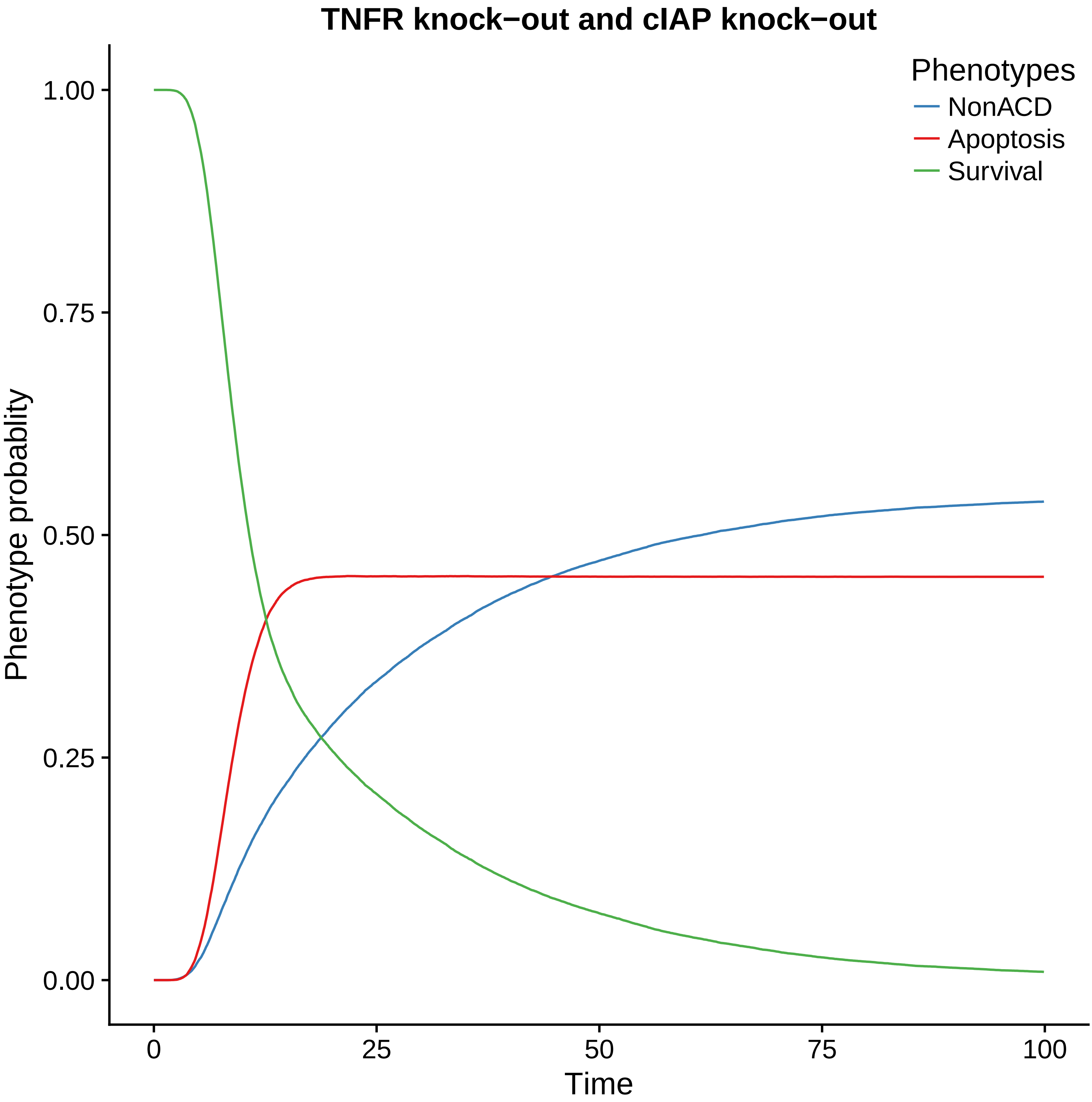

TNFR knock−out and cIAP knock−out
Phenotypes
NonACD
Apoptosis
Survival
Phenotype probablity
1.00
0.75
0.50
0.25
0.00
0
25
50
75
100
Time
