# Supplementary material for: PhysiBoSS: a multi-scale agent-based modelling framework integrating physical dimension and cell signalling
Source: Bioinformatics. 2018 Aug 30;35(7):1188–96. doi: 10.1093/bioinformatics/bty766 (PMC6449758; doi:10.1093/bioinformatics/bty766)
Supplement: Supplementary Information [file bty766_supplementary_information.pdf]

Supplementary Informations

Supplementary informations for PhysiBoSS: a multi-scale agent based modelling framework integrating physical dimension and cell signalling

Gaelle Letort, Arnau Montagud, Gautier Stoll, Randy Heiland, Emmanuel Barillot, Paul Macklin, Andrei Zinovyev, Laurence Calzone

Contents

1 Figure S1: PhysiBoSS features **2**

2 Figure S2: Cell fate decision Boolean model **3**

3 Figure S3: Population response to TNF injection **4**

4 Figure S4: Effects of simulation parameters in spheroids. **5**

5 Figure S5: Genetically heterogeneous population under TNF treatment. **7**

6 Figure S6: Genetically heterogeneous population under TNF treatment and oxygen-limited regime. **8**

7 Table S1: Different multi-scale modelling frameworks **10**

8 Table S2: Simulation run times **12**

1 Figure S1: PhysiBoSS features

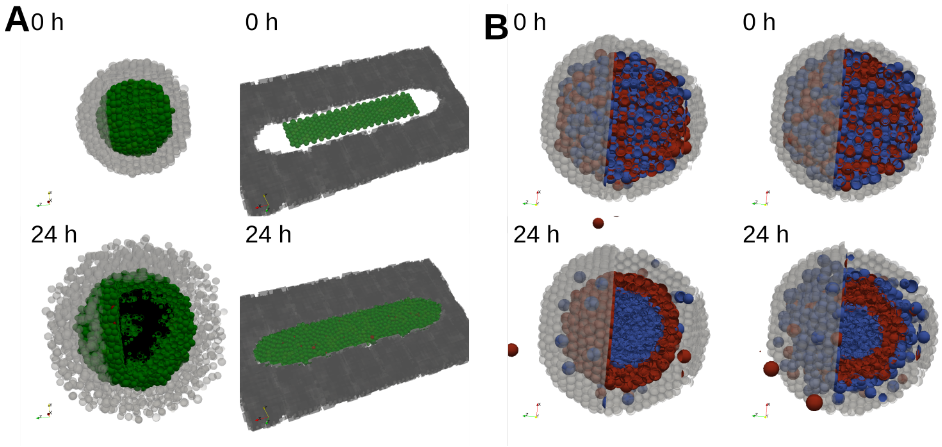

Initial and final snapshots of the simulations presented in main text, Figure 2.

2 Figure S2: Cell fate decision Boolean model

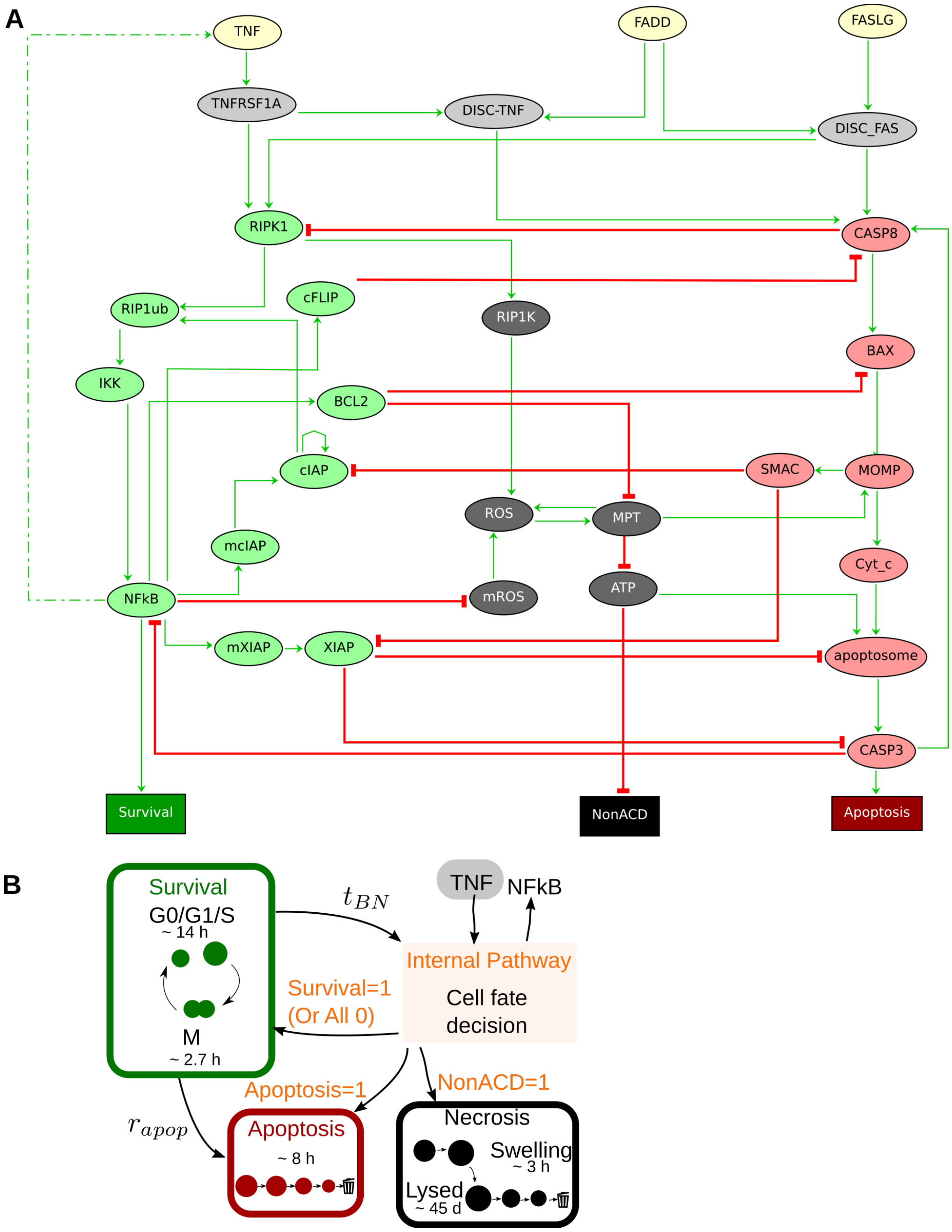

A: Boolean network used. Pathway related to *Survival* (green), related to *Apoptosis* (red) and related to *NonACD* (black). Green arrows represent activation, red arrows inhibitions. B: Schematic representation of how cell cycle is simulated. Cells are initially in *Proliferative* state (growing and dividing; green). At frequent interval, their internal signalling network is updated by running MaBoSS (orange) given its environmental conditions (for instance, internalization of TNF). These conditions affects the fate of the cell: to continue proliferating or commit to *Apoptosis* (red) or *NonACD* (black).

3 Figure S3: Population response to TNF injection

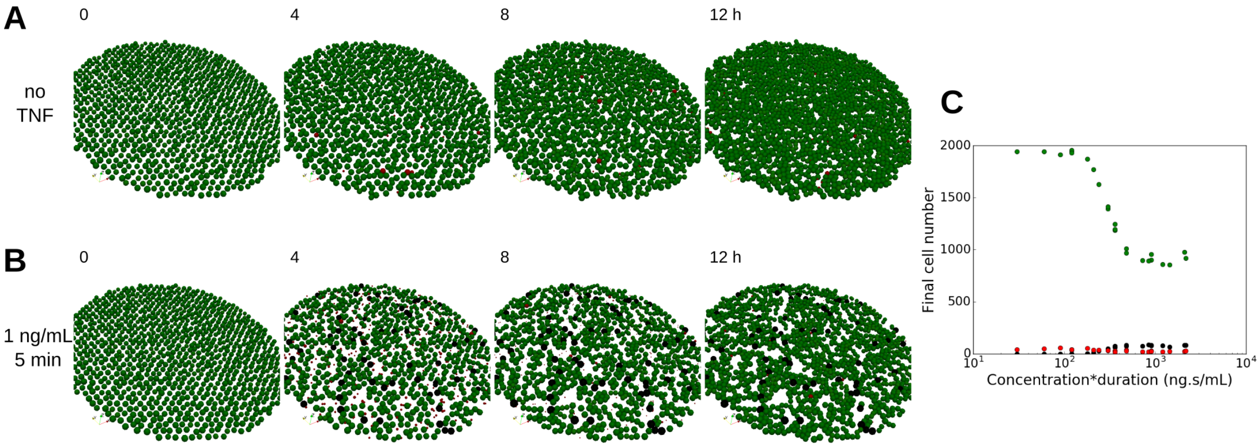

Supporting informations for main text, Figure 3. A: Simulations without TNF. Snapshots of a simulation at t = 0, 4, 8 and 12h. B: Simulations under a low dose injection of TNF (1 ng/mL during 5 min). Snapshots of a simulation at t = 0, 4, 8 and 12h. C: Final fate of the active cells in the simulation for each 'stimulus area' tested. A-C: Colour code: green, *Proliferative* cells; red, cells committed to *Apoptosis*; black, cells committed to *NonACD*.

4 Figure S4: Effects of simulation parameters in spheroids.

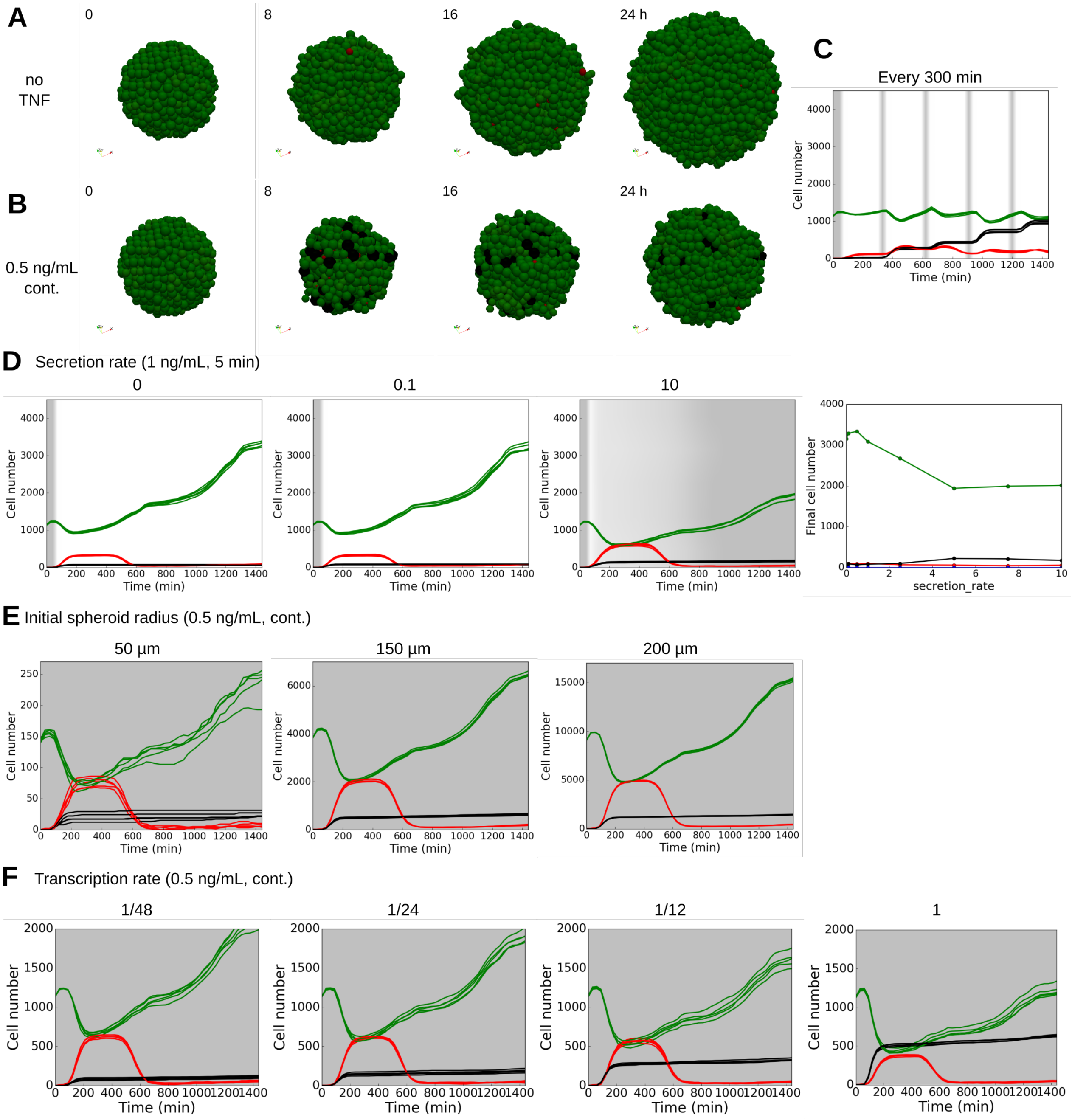

A: Growth without TNF. Snapshots of a simulation of a spheroid growth at t = 0, 8, 16 and 24 h. B: Growth under a 0.5 ng/mL continuous dose of TNF. Snapshots of a simulation at t = 0, 8, 16 and 24 h. C: Growth under a pulsed injection of TNF, 0.5 ng/mL for 5 min every 300 min. D: Variation of secretion rate value. Time evolution of the cell population in each fate (three leftmost panels) for a secretion rate of: 0 (1st panel), 0.1 (2nd) and 10 (3rd). TNF was injected at the beginning of the simulation for 5 min at a dose of 1 ng/mL. Note that when secretion rate

is 10, TNF accumulates in the environment (grey shading). Final number of cells in each fate according to the secretion rate used for individual simulations (rightmost panel). E: Variation of the initial spheroid radius. Time evolution of the cell population in each cell fate for an initial population radius of 50 (left panel), 150 (middle) and 200 (right) μm. TNF is injected continuously during the simulations at a concentration of 0.5 ng/mL. In order to better show population dynamics, Y-axes are different among panels. F: Variation of the transcription rate used in the Boolean network transitions. Time evolution of the cell population in each cell fate for a transcription rate of 1/48 (1st panel), 1/24 (2nd, the one used in all other results), 1/12 (3rd) and 1 (4th panel). Transitions rates for other nodes described in the note below are 1. A-F: Colour code: green, *Proliferative* cells; red, cells committed to *Apoptosis*; black, cells committed to *NonACD*. Grey shading indicates TNF presence. Except in D, right panel, 5 simulations are presented for each condition. Simulation time is 24 h, initial spheroid radius is 100 μm, which accounts for roughly 1000 cells, except explicitly stated.

Note on the different rates of transition

Regarding the different rates of transition used in our model, two sets of nodes were established: first, most of the nodes present in the model represent protein-to-protein interactions and signal transduction such as *Apoptosis* pathway activation, TNF activation of *Survival* pathway and ROS activation. The value for these nodes was 1 MaBoSS rate that corresponds roughly to 10 minutes of PhysiBoSS time. Additionally, a subset of nodes, the ones that represent the activation of BCL2 and cFLIP as well as translation of different mRNAs (mROS, mcIAP and mXIAP) (all of them activated by NFkB) and the translation of cIAP from mcIAP, were considered to be activated in much more time (1/24 MaBoSS rate that corresponds roughly to 4 hours of PhysiBoSS time).

We considered that protein-to-protein interactions and signal transduction happened 24 times faster than the transcriptional and translation steps. This was an educated guess based in previous works: Hirata *et al.* (2002) and Shimojo *et al.* (2008) found that transcription is 12 times faster than translation in mouse cells (and Lewis (2003) considered this as well for zebrafish). In our case, most interactions are protein activations, and those are faster than translation, therefore considering a factor of 24 between protein activations and translation.

Note on spheroid size comparison to experiments

In Figure 4A, we present simulations without TNF injections: spheroids with an initial radius of 100 μm with ≈1000 cells reached a size of approximatively 150 μm radius with ≈4500 cells in 24h, a growth of 4.5x factor. In this case, these results consider that oxygen is freely available to cells, even in the centre of the spheroid. Additionally, in Figure S6A in Supplementary Information we present results without TNF injection and with oxygen and nutrients limitations, a closer set-up to experimental data. In this case, an initial 9000-cells spheroid has a 200 μm radius and after 24 hours grows to a final ≈16500 cells spheroids with a 245 μm radius, a growth of 1.8x factor.

Experimentally, comparable population growths have been observed in Freyer *et al.* (1984) work, which measured a fivefold increase in two spheroids (from 1000 to 5000 cells in one and from 600 to 3000 cells in another). Nonetheless, the number of cells in the spheroids are quite different, which is likely due to the different cell strains we are comparing: V-79 Chinese hamster fibroblasts in Freyer *et al.* (1984) and 3T3 Swiss albino mouse embryo fibroblasts simulated in present work with data taken from Tay *et al.* (2010) and Kellogg *et al.* (2015). This density discrepancy is also a probable cause for the differences we see in terms of volume changes. Freyer *et al.* (1984) 1000-cells spheroid's radius goes from 210 μm to a final 230 μm for the 5000-cells spheroid (9.5% radius increase) and 600-cells spheroid's radius goes from 150 μm to a final 220 μm for the 3000-cells spheroid (32% radius increase), while our simulated spheroid has a 50% radius increase without oxygen limitation. However, in simulations with oxygen and nutrient limitations, we observed that these are impeding factors for growth once the spheroid goes

beyond a threshold volume. Indeed, considering these limitations, we measured a radius increase of approximatively 25%, which is in range of the experimental values. These considerations highlight the importance of multi-scale frameworks that take into account both environmental and genetic information.

5 Figure S5: Genetically heterogeneous population under TNF treatment.

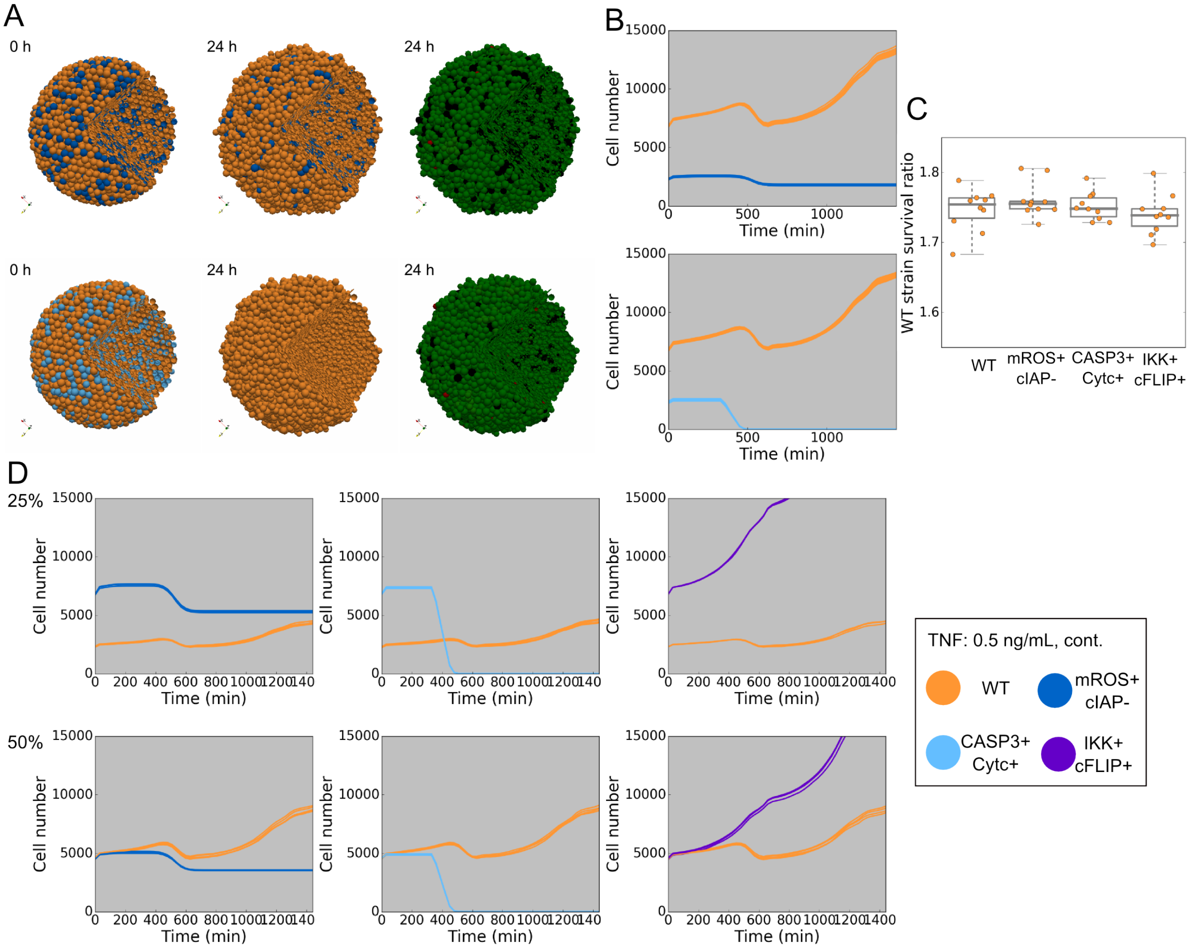

Simulations of heterogeneous population composed of 75% of WT cells (orange) and 25% of mROS+ and cIAP- mutated cells (blue) or with CASP3+ and Cytc+ mutated cells (light blue). A: Snapshots of a simulation for each case at initial and final time (24 h), with cells coloured by cell type (left and middle) or by cell fate commitment (right). B: Time evolution of the number of cells of each strain (WT and mutated) for 10 simulations. C: Ratio of final number of surviving cells against initial number of cells for each cell line (WT or mutated). No significant differences were found under Kolmogorov-Smirnov test. D: Time evolution of the number of cells in each strain (WT and mutated) for 10 simulations for the 3 different mutants with different initial proportion of WT cells compared to the total population: 25% (top) and 50% (bottom). A-C: Colour code: green, *Proliferative* cells; red, cells committed to *Apoptosis*; black, cells committed to *NonACD*. Grey shading indicates presence of TNF in continuous injection at 0.5 ng/mL. Initial spheroid radius is 200 μm, which accounts for roughly 9000 cells, + stands for

over-expression and - stands for knock-out.

6 Figure S6: Genetically heterogeneous population under TNF treatment and oxygen-limited regime.

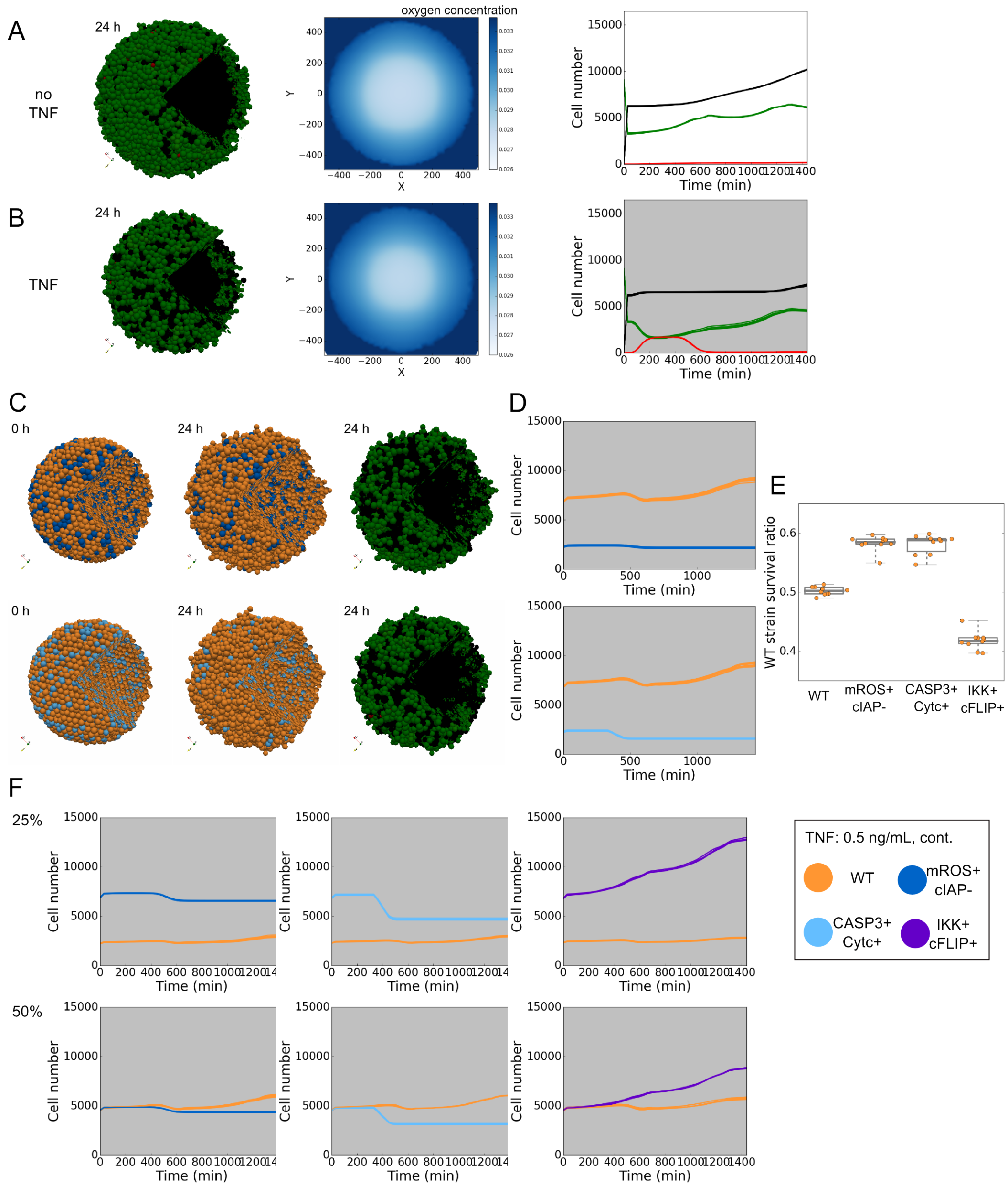

A: Simulation of WT-only population without TNF. Snapshots of a simulation (left) and oxygen levels (middle) at 24 h. Time evolution of the number of cells in each cell fate (right). B: Simulation of WT-only population for a low-dose injection of TNF. Snapshots of a simulation (left) and oxygen levels (middle) at 24 h. Time evolution of the number of cells in each cell fate (right). C-E: Simulations of heterogeneous population and TNF treatment composed of 75% of WT cells (orange) and 25% of mROS+ and cIAP- mutated cells (blue) or with CASP3+ and Cytc+ mutated cells (light blue). C: Snapshots of a simulation for each case at initial and final

time (24 h), with cells coloured by cell type (left and middle) or by cell fate commitment (right). D: Time evolution of the number of cells in each strain (WT and mutated) for 10 simulations. E: Ratio of final number of surviving cells against initial number of cells for each cell line (WT or mutated). F: Time evolution of the number of cells in each strain (WT and mutated) for 10 simulations for the 3 different mutants with different initial proportion of WT cells compared to the total population: 25% (top) and 50% (bottom). A-F: Oxygen levels, represented from dark blue (injected level) to white (lowest level), are measured in the z=0 plane of the simulated space. Colour code: green, *Proliferative* cells; red, cells committed to *Apoptosis*; black, cells committed to *NonACD*. Grey level background in graphs indicates presence of TNF in continuous injection at 0.5 ng/mL. All simulations are in oxygen-limited regime, initial spheroid radius is 200 μm, which accounts for roughly 9000 cells, + stands for over-expression and - stands for knock-out.

7 Table S1: Different multi-scale modelling frameworks

Examples of different multi-scale modelling frameworks that could be used to address in different manners problems similar to the ones presented in this work. Intracellular framework can be differential equations, ordinary (ODE) or partial (PDE), or logical modelling (Boolean or multilevel). Extracellular framework can be based on a lattice (cellular automata (CA) or Cellular Potts (CP)) or independent from a lattice description (agent-based (AB) or vertex-based (VB)). Dimensions (Dim.) can be 1: 1D (lines), 2: 2D (areas), 3: 3D (volumes) or 3m: 3D objects in a monolayer. OS compatibility can be Linux (L), Mac OS (M) or Windows (W).

Name, ref.	Intracellular framework	Extracellular framework	Cell shape	Dim.	OS	Parallelization	Code license (and link)
Athale and Deisboeck (2006)	ODE	off-lattice AB	squares	2	L	Not available	Not available
CellSys<br>Hoehme and Drasdo (2010)	ODE	off-lattice AB	spheres	1,2, 3,3m	L	OpenMP	Propietary
Chaste<br>Mirams *et al.* (2013)	ODE, PDE	off-lattice (AB, VB) and on-lattice (CA, CP)	spheres	1,2, 3,3m	L,M,W	OpenMP, MPI	Open
EmbryoMaker<br>Marin-Riera *et al.* (2016)	ODE	off-lattice AB	spheres, cylinders	3,3m	L,M	Not available	Open
EpiLog<br>Varela *et al.* (2018)	Logical	on-lattice CA	hexagons	2	L,M,W	Not available	Not available
MecaGen	ODE	off-lattice AB	spheres	3,3m	L	OpenMOLE compatible	Open
Orchestral<br>Coulier and Hellander (2018)	ODE	depends on single-cell modelling	depends on single-cell modelling	depends on single-cell modelling	L,M,W	Dask	Open
PhysiBoSS	Logical	off-lattice AB	spheres	3,3m	L,M, W(docker)	OpenMP	Open
Prokopiou *et al.* (2014)	ODE	on-lattice CP	squares can be used to form other shapes	2	L,M,W	CompuCell3D is OpenMP compatible	Uses CompuCell3D
Schluter *et al.* (2014)	ODE	off-lattice AB	spheres	3m	-	Not available	Not available
VirtualLeaf<br>Merks *et al.* (2011)	ODE	off-lattice VB	irregular	2	L,M,W	Not available	Open

8 Table S2: Simulation run times

Representative examples of run time necessary for different kind of simulations. Simulations were run on one node of a Linux cluster (with 16 OpenMP threads). The processes used around 300 MB of memory while running (Proportional Set Size measured, from 284 MB for 1000 cells to 295 MB for 10000 cells).

Cell number (init-final)	Simulated time	Remark	Wall time
1022 - 1969	720 min	no TNF	2 min
1024 - 1443	720 min	cont. TNF	1.5 min
1131 - 4772	1440 min	no TNF	10.5 min
1134 - 2211	1440 min	cont. TNF	25.75 min
1136 - 3544	1440 min	one pulse injection	9 min
3843 - 7284	1440 min	cont. TNF	42 min
16297 - 23227	1440 min	7192 passive cells	45.5 min

References

Athale, C. A. and Deisboeck, T. S. (2006). The effects of egf-receptor density on multiscale tumor growth patterns. *Journal of Theoretical Biology*, **238**(4), 771–779.

Coulier, A. and Hellander, A. (2018). Orchestral: a lightweight framework for parallel simulations of cell-cell communication. *arXiv:1806.10889 [q-bio.CB]*.

Freyer, J. P., Tustanoff, E., Franko, A. J., and Sutherland, R. M. (1984). In situ oxygen consumption rates of cells in v-79 multicellular spheroids during growth. *Journal of Cellular Physiology*, **118**(1), 53–61.

Hirata, H., Yoshiura, S., Ohtsuka, T., Bessho, Y., Harada, T., Yoshikawa, K., and Kageyama, R. (2002). Oscillatory Expression of the bHLH Factor Hes1 Regulated by a Negative Feedback Loop. *Science*, **298**(5594), 840–843.

Hoehme, S. and Drasdo, D. (2010). Biomechanical and Nutrient Controls in the Growth of Mammalian Cell Populations. *Mathematical Population Studies*, **17**(3), 166–187.

Kellogg, R. A., Tian, C., Lipniacki, T., Quake, S. R., and Tay, S. (2015). Digital signaling decouples activation probability and population heterogeneity. *eLife*, **4**(OCTOBER2015), 1–26.

Lewis, J. (2003). Autoinhibition with Transcriptional Delay. *Current Biology*, **13**(16), 1398–1408.

Marin-Riera, M., Brun-Usan, M., Zimm, R., Välikangas, T., and Salazar-Ciudad, I. (2016). Computational modeling of development by epithelia, mesenchyme and their interactions: a unified model. *Bioinformatics (Oxford, England)*, **32**(2), 219–25.

Merks, R. M. H., Guravage, M., Inzé, D., and Beemster, G. T. S. (2011). Virtualleaf: An open-source framework for cell-based modeling of plant tissue growth and development. *Plant Physiology*, **155**(2), 656–666.

Mirams, G. R., Arthurs, C. J., Bernabeu, M. O., Bordas, R., Cooper, J., Corrias, A., Davit, Y., Dunn, S. J., Fletcher, A. G., Harvey, D. G., Marsh, M. E., Osborne, J. M., Pathmanathan, P., Pitt-Francis, J., Southern, J., Zemzemi, N., and Gavaghan, D. J. (2013). Chaste: An Open Source C++ Library for Computational Physiology and Biology. *PLoS Computational Biology*, **9**(3).

Prokopiou, S. A., Barbarroux, L., Bernard, S., Mafille, J., Leverrier, Y., Arpin, C., Marvel, J., Gandrillon, O., and Crauste, F. (2014). Multiscale modeling of the early cd8 t-cell immune response in lymph nodes: An integrative study. *Computation*, **2**(4), 159–181.

Schluter, D. K., Ramis-Conde, I., and Chaplain, M. A. J. (2014). Multi-scale modelling of the dynamics of cell colonies: insights into cell-adhesion forces and cancer invasion from in silico simulations. *Journal of The Royal Society Interface*, **12**(103), 20141080–20141080.

Shimojo, H., Ohtsuka, T., and Kageyama, R. (2008). Oscillations in Notch Signaling Regulate Maintenance of Neural Progenitors. *Neuron*, **58**(1), 52–64.

Tay, S., Hughey, J. J., Lee, T. K., Lipniacki, T., Quake, S. R., and Covert, M. W. (2010). Single-cell NF-κB dynamics reveal digital activation and analogue information processing. *Nature*, **466**(7303), 267–271.

Varela, P. L., Monteiro, P. T., Mendes, N. D., Fauré, A., and Chaouiya, C. (2018). Epilog: A software for the logical modelling of epithelial dynamics [version 1; referees: awaiting peer review]. *F1000Research*, **7**, 1145.
